# Derivatives of Phenyl Pyrimidine and of the Different Donor Moieties as Emitters for OLEDs

**DOI:** 10.3390/ma17061357

**Published:** 2024-03-15

**Authors:** Hryhorii Starykov, Oleksandr Bezvikonnyi, Karolis Leitonas, Jurate Simokaitiene, Dmytro Volyniuk, Eigirdas Skuodis, Rasa Keruckiene, Juozas Vidas Grazulevicius

**Affiliations:** 1 Department of Polymer Chemistry and Technology, Faculty of Chemical Technology, Kaunas University of Technology, K. Barsausko St. 59, LT-51423 Kaunas, Lithuania; hryhorii.starykov@ktu.edu (H.S.); o.bezvikonnyi@ktu.lt (O.B.); karolis.leitonas@ktu.lt (K.L.); jurate.simokaitiene@ktu.lt (J.S.); dmytro.volyniuk@ktu.lt (D.V.); eigirdas.skuodis@ktu.lt (E.S.); rasa.keruckiene@ktu.lt (R.K.); 2 Department of Physics, Faculty of Mathematics and Natural Sciences, Kaunas University of Technology, Studentų St. 50, LT-51369 Kaunas, Lithuania

**Keywords:** phenyl pyrimidine, triphenylamine, methoxy group, TADF, OLED

## Abstract

Two derivatives of phenyl pyrimidine as acceptor unit and triphenylamino or 4,4′-dimethoxytriphenylamino donor groups were designed and synthesized as emitters for organic light-emitting diodes (OLEDs) aiming to utilize triplet excitons in the electroluminescence. Thermogravimetric analysis revealed high thermal stability of the compounds with 5% weight loss temperatures of 397 and 438 °C. The theoretical estimations and photophysical data show the contributions of local excited and charge transfer states into emission. The addition of the methoxy groups led to the significant improvement of hole-transporting properties and the bathochromic shift of the emission from blue to green-blue spectral diapason. It is shown that mixing of the compounds with the organic host results in facilitation of the delayed emission. The singlet–triplet energy splitting was found to be too big for the thermally activated delayed fluorescence. No thermal activation of the long-lived emission was detected. No experimental evidence for triplet–triplet annihilation and room temperature phosphorescence were detected making the hot exciton mechanism the most probable one. The OLEDs based on the compounds reached the maximum external quantum efficiency of up to 10.6%.

## 1. Introduction

Search for new materials with advanced properties is one of the major tasks of the rapidly developing field of organic optoelectronics. The technology of organic light-emitting diodes (OLED) has undergone rapid development in recent years [1,2]. Great effort was put into solving the problem of the efficient utilization of triplet excitons in electroluminescence (EL). Singlet and triplet excitons are formed under the applied voltage in the ratio of 1 to 3 due to spin statistics [3]. The radiative deactivation of triplet excitons via phosphorescence involves a transition to the ground state of different multiplicity making the transition forbidden from the point of view of quantum mechanics [4]. The stimulation of phosphorescence in OLEDs was achieved by the application of noble metal complexes as emitters [5,6]. Usage of the complexes of noble metals such as Ir, Pt, and Au, significantly increases the cost of production of phosphorescent OLEDs (PhOLEDs) and raises environmental issues [7]. The phenomenon of room temperature phosphorescence (RTP) is exploited in order to solve the problem of the high cost. The principle of RTP is based on the facilitation of spin–orbit interactions by the presence of heavy atoms like S, Br, P, I, Te, etc., in the structures of emitters utilizing *d*-electrons [8]. Other approaches were developed to compete the phosphorescence as a path for the exploitation of the emission of triplet excitons. Thermally activated delayed fluorescence (TADF) is known as the delayed emission of upconverted triplet excitons that are deactivated from the single excited states [9]. When donating and accepting units are separated spatially within a molecule of organic emitter or of two different molecules, intra- or inter-molecular charge transfer, respectively, occurs. It is accompanied by a decrease in the difference between the energy levels of the first singlet and triplet excited states [10]. Taking this into account, TADF can be also achieved for exciplex-forming molecular mixtures [11]. The thermal motion of atoms can cause reverse intersystem crossing (RISC) if the singlet–triplet energy splitting is sufficiently small [12]. In some cases, RISC can occur at higher energy levels than the first excited states. In such a case, the hot excitons migrate to the singlet excited states followed by relaxation and fluorescence [13]. Hybridized local and charge-transfer excited state (HLCT) is utilized in RISC providing an alternative to TADF [14]. Another phenomenon that allows the utilization of triplet excitons is triplet–triplet annihilation (TTA) [15]. Two triplet excitons can annihilate forming a single singlet exciton of twice higher energy, which can emit via deactivation or additionally through the relaxation to the lowest singlet excited state [16]. The major drawback of TTA is that the two-exciton process limits the maximum internal quantum efficiency (IQE) of OLEDs to 67.5%. Meanwhile, RTP can allow the IQE of the devices to reach 100% [17]. An IQE of 100% was already reported for TADF [18] and phosphorescent [17] OLEDs.

The usage of aromatic amines as electron-donating groups in the design of organic emitters can assist in the improvement of hole-transporting properties, through the formation of high-stability cation radical species [19]. The distorted propeller-like geometry of triphenylamino groups allows it to hinder intermolecular interactions and stimulate fluorescence.

Pyrimidine derivatives are widely used in organic electronic devices, such as OLEDs, solar cells, sensors, etc. [20,21,22,23]. The C=N double bond of pyrimidine makes it a strong electron acceptor. Utilizing this property, pyrimidine was incorporated into structures of various fluorescent, phosphorescent emitters, bipolar hosts, and functional materials for electron transport layers [20]. Pyrimidine was used as a building block of compounds capable of utilization of triplet excitons in emission including TADF emitters [24,25,26,27,28], and phosphorescent emitters for PhOLEDs [29,30,31]. Deep blue emitters possessing the HLCT property having carbazoles as donors and 9,9-dimethylfluorenyl and spirofluorenyl groups forming extended accepting moieties with the pyrimidine unit were reported to be utilized in the doping-free emitting layers (EML) of OLEDs with the maximum external quantum efficiency of 5.9–6.6% [32]. Our group recently reported the pyrimidine-5-carbonitriles with triphenylamino- or 9-phenyl carbazolyl groups, which exhibited the upper RISC of the hot excitons [33]. The compounds were utilized as emitters of blue/sky blue OLEDs reaching EQE of up to 7% and as hosts for orange-red TADF-based OLEDs with EQE of up to 20.7%.

In this work, the compounds with a pyrimidine acceptor moiety and the different donor moieties suitable for OLEDs were synthesized and studied. Organic semiconductors with a pyrimidine acceptor moiety and triphenylamino or 4,4′-dimethoxytriphenylamine donor moieties were obtained after a two-step synthesis. The compound without methoxy groups was reported before and synthesized using different synthetic routes [34]. The presented theoretical and experimental data in our study are consistent with the previously reported findings. The compounds exhibit the property of reverse intersystem crossing (RISC) utilizing the states with higher energy than the first excited states. This property was observed when the compounds were doped in an organic matrix. The electronic excitation energy transfer from host to guest resulted in the appearance of long-lived emission and consequent increase in the EQE of OLED up to 10.6% proving the utilization of triplet excitons.

## 2. Experimental Section

### 2.1. Materials and Methods

4,4-Dichlorochalcone, 4,4′-dimethoxyphenylamine, palladium(II) acetate, tri-*tert*-butylphosphine, 4-benzamidine hydrochloride hydrate, diphenylamine, sodium *tert*-butoxide, sodium chloride, potassium hydroxide were purchased from Sigma Aldrich (St. Louis, MO, USA) or Fluorochem (Hadfield, UK) and used as received.

#### 2.1.1. 4,6-Bis(4-Chlorophenyl)-2-phenylpyrimidine

4,4-Dichlorchalcone (1.509 g, 5.43 mmol) and 4-benzamidine hydrochloride hydrate (0.869 g, 4.980 mmol) were dissolved in ethanol (25 mL). The mixture was stirred and heated to reflux. After 15 min, 3M aqueous solution of potassium hydroxide (0.869 g, 15.44 mmol) was added to the mixture dropwise. At the end of the reaction, the resulting mixture was extracted with ethyl acetate/water, and organic layer was washed with saturated brine solution, dried over Na_2_SO_4_, and concentrated under reduced pressure. The product was recrystallized from methanol. The yield of white crystals was 20% (0.30 g). M_p_ = 198–200 °C.

^1^H NMR (400 MHz, CDCl_3_) δ, ppm: 8.71–8.64 (m, 2H); 8.21 (d, 4H, *J* = 8.1 Hz); 7.90 (s, 1H), 7.58–7.48 (m, 7H).

^13^C NMR (101 MHz, CDCl_3_) δ, ppm: 164.62, 163.72, 137.71, 137.18, 135.71, 130.94, 129.20, 128.58, 128.54, 128.48, 109.66.

IR ν_max_ (KBr), cm^−1^: 3038, 1584, 1566, 1523, 1488, 1402, 925, 751, 683.

Calcd. C_22_H_14_Cl_2_ N_2_ [M]^+^ exact mass 376.05 found MS (ES^+^) 376.97 [M]^+^, 378.97 [M+2]^+^.

#### 2.1.2. 4,6-Bis(4-Diphenylamino-phenyl)-2-phenylpyrimidine (PP1)

The mixture of 4,6-bis(4-chlorophenyl)-2-phenylpyrimidine (0.2 g, 0.530 mmol), diphenylamine (0.188 g, 1.113 mmol), tri-*tert*-butylphosphine (0.05 mL, 0.04 mmol), palladium(II) acetate (0.005 g, 0.04 mmol) and sodium *tert*-butoxide (0.280 g, 2.915 mmol) in toluene (10 mL) was stirred at 110 °C under inert atmosphere for 24 h. When the reaction was finished, the mixture was extracted with ethyl acetate/water, and organic layer was washed with saturated brine solution, dried over Na_2_SO_4_, and concentrated under reduced pressure. The product was purified by column chromatography on silica gel using a mixture of hexane and acetone in a volume ratio of 10:1 as the eluent. The product was recrystallized from hexane. The yield of white crystals was 85% (0.17 g). M_p_ = 249–250 °C.

^1^H NMR (400 MHz, CDCl_3_) δ, ppm: 8.69 (d, 2H, *J* = 7.2 Hz); 8.15 (d, 4H, *J* = 8.4 Hz); 7.85 (s, 1H), 7.56–7.46 (m, 3H); 7.32 (t, 8H, *J* = 7.6 Hz); 7.19 (d, 12H, *J* = 7.8 Hz); 7.11 (t, 4H, *J* = 7.2 Hz).

^13^C NMR (101 MHz, CDCl_3_) δ, ppm: 164.10, 163.81, 150.35, 147.14, 138.25, 130.50, 130.46, 129.48, 128.46, 128.38, 128.26, 125.28, 123.85, 122.13, 108.59.

IR ν_max_ (KBr): 3037, 1586, 1564, 1504, 1486, 1418, 900, 753, 694.

Calcd. C_46_H_34_ N_4_ [M]^+^ exact mass 642.28 found MS (ES^+^) 643.14 [M+1] ^+^.

#### 2.1.3. 4,6-Bis((4-Di(4-metoxyphenil)amino)-phenil)-2-phenilpyrimidine (PP2)

A mixture of 4,6-bis(4-chlorophenyl)-2-phenylpyrimidine (0.700 g, 1.855 mmol), 4,4′-di(methoxyphenyl)amine (0.893 g, 3.896 mmol), tri-*tert*-butylphosphine (0.15 mL, 0.074 mmol), palladium(II) acetate (0.017 g, 0.074 mmol) and sodium *tert*-butoxide (0.981 g, 10.250 mmol) in toluene (35 mL) was stirred at 110 °C under inert atmosphere for 24 h. The mixture was extracted with ethyl acetate/water, and organic layer was washed with saturated brine solution, dried over Na_2_SO_4_, and concentrated under reduced pressure. The product was purified by column chromatography on silica gel using hexane and acetone as the eluent mixture of solvents in a volume ratio of 6:1. The product was recrystallized from hexane. The yield of yellow crystals was 64% (0.45 g). M_p_ = 213–215 °C.

^1^H NMR (400 MHz, CDCl_3_) δ, ppm: 8.67 (d, 2H, *J* = 6.0 Hz); 8.09 (d, 4H, *J* = 8.7 Hz); 7.79 (s, 1H), 7.55–7.41 (m, 3H); 7.13 (d, 8H, *J* = 8.8 Hz); 7.02 (d, 4H, *J* = 8.6 Hz); 6.88 (d, 8H, *J* = 8.8 Hz), 3.82 (s, 12H).

^13^C NMR (101 MHz, CDCl_3_) δ, ppm: 163.91, 163.73, 156.48, 151.09, 140.13, 138.43, 130.35, 128.64, 128.43, 128.31, 128.12, 127.28, 119.15, 114.85, 108.00, 55.53.

IR ν_max_ (KBr): 3045, 2832, 1602, 1585, 1561, 1400, 912, 756, 700.

Calcd. C_50_H_42_ N_4_O_4_ [M]^+^ exact mass 762.32 found MS (ES^+^) 762.83 [M]^+^.

## 3. Results and Discussion

### 3.1. Synthesis and Thermal Properties

**Scheme 1:**
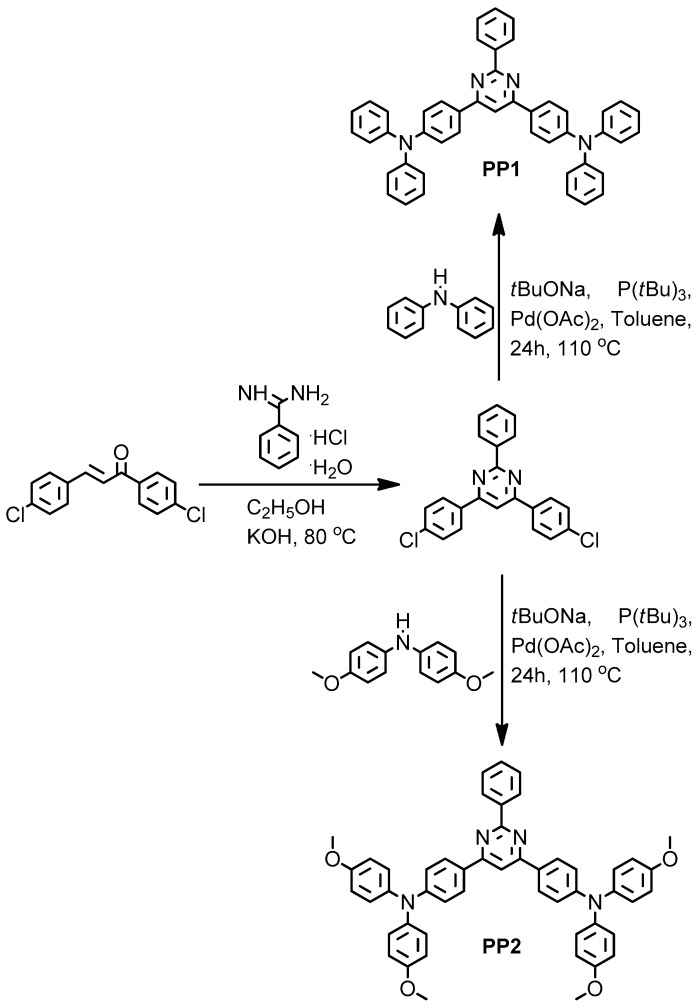
Synthesis of compounds PP1 and PP2.

Derivatives of 2-phenylpyrimidine containing triphenylamino or 4,4′-dimethoxy triphenylamino moieties were synthesized with relatively high yields (Scheme 1). Firstly, the parent 4,6-bis(4-chlorophenyl)-2-pheniypyrimidine was obtained by reaction of phenyl amidine with 4,4′-dichlorochalcone in the presence of KOH. In the second step, diphenylamino or 4,4′-di(methoxyphenyl)amino fragments were attached via the Buchwald–Hartwig amination reaction to achieve the targeted compounds PP1 and PP2. The structure of the compounds was confirmed by ^1^H and ^13^C NMR, IR, and mass spectrometry.

**Figure 1:**
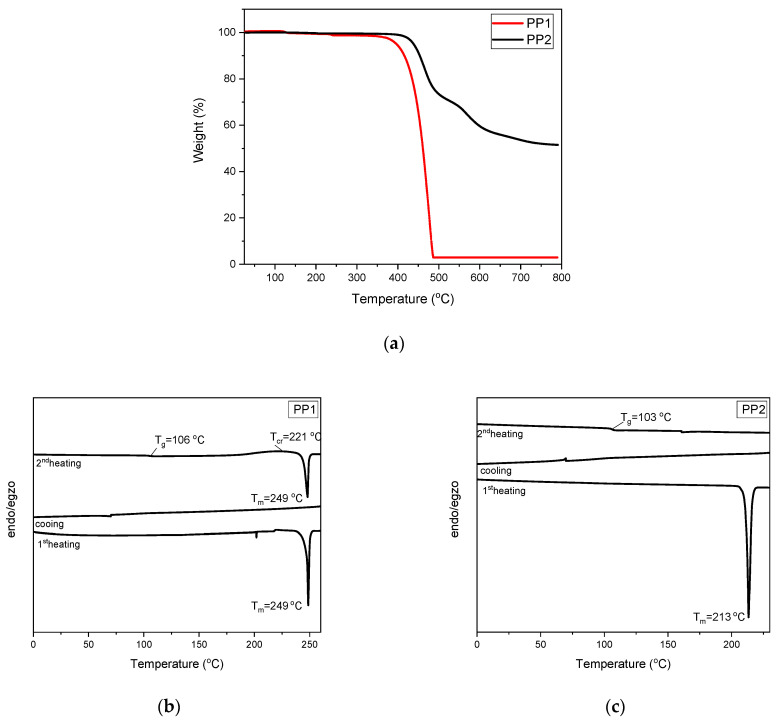
TGA curves of compounds PP1 and PP2 (**a**). DSC curves of compounds PP1 (**b**) and PP2 (**c**).

The thermal properties of the target compounds were investigated by thermogravimetric analysis (TGA) and differential scanning calorimetry (DSC). The obtained thermograms are presented in Figure 1. The data are collected in Table 1.

The value of 5% weight loss temperature (*T_ID_*_-5%_) of compound PP2 was found to be 438 °C. Whereas the TGA curve of compound PP1 shows a sublimation process upon heating at approximately 397 °C. The compounds are characterized by high thermal stability and are suitable for layer formation by vacuum evaporation.

Using differential scanning calorimetry, the morphological changes in the compounds were studied. Compounds PP1 and PP2 were obtained as crystalline substances after the synthesis. The melting temperatures of PP1 and PP2 were determined from the DSC thermograms of the first heatings at 249 and 213 °C, respectively. Both the compounds formed molecular glasses. The glass transition temperatures of PP1 and PP2 were found to be 106 and 103 °C, respectively. The molecular glass of compound PP2 was found to be morphologically more stable than that of PP1. Neither crystallization nor melting signals were recorded in the DSC thermogram of the second heating of PP2. In contrast, the molecular glass of compound PP1 in the second heating showed glass transition, crystallization, and melting signals at 106, 221, and 249 °C, respectively.

### 3.2. Theoretical Calculations, Electrochemical, and Photoelectric Properties

**Figure 2:**
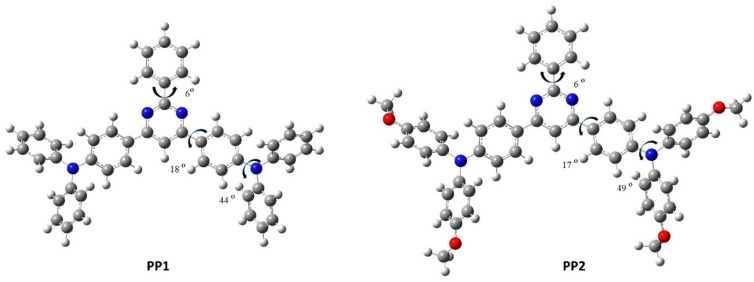
Optimized geometries of PP1 and PP2 at the ground state, calculated at B3LYP/6-31G (d,p) level. (Grey colour: carbon; blue: nitrogen; red: oxygen; white: hydrogen).

Geometry optimization of the compound structures was performed using the density functional theory (DFT). The ground state was optimized at the B3LYP/6-31G (d, p) level in the gas phase (Figure 2). The B3LYP/6-31G (d, p) split-valence double-zeta basis set is used as a standard to calculate the electronic wavefunctions of the molecular systems [35,36]. As the pyrimidine acceptor and donor diphenylamine moieties are linked through a phenyl bridge, the values of dihedral angles of these moieties are pertinent for the intermolecular charge transfer evaluation. In the case of the investigated compounds PP1 and PP2, the dihedral angle values determined from the optimized geometries are quite low, indicating a quite extended π-conjugated electron system.

**Figure 3:**
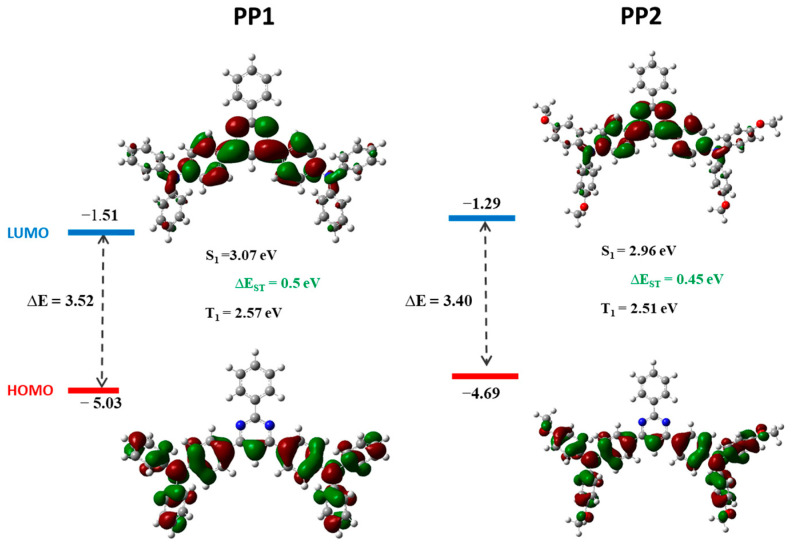
DFT calculated HOMO, LUMO energies, and their topologies (isovalue of 0.02) of compounds PP1 and PP2. (Grey colour: carbon; blue: nitrogen; red: oxygen; white: hydrogen).

The calculated highest occupied molecular orbital (HOMO) and lowest unoccupied molecular orbital (LUMO) values and visualizations are presented in Figure 3. The quite flat core of the molecular structures of compounds PP1 and PP2 translates to HOMO delocalization over the diphenylamine donors and central pyrimidine acceptor. As for the LUMOs, they are delocalized on the acceptor moiety and the phenyl bridges. Such electronic structures of compounds PP1 and PP2, with the overlap of orbitals, would heavily influence the absorption and emission properties.

**Figure 4:**
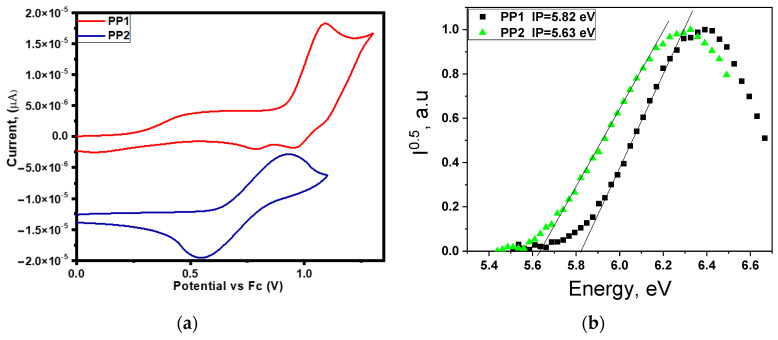
Cyclic voltammograms of the solutions of PP1 and PP2 (**a**) in 0.1 M TBAPF_6_/dichloromethane solutions recorded at scan rate of 0.1 mVs^−1^. Electron photoemission spectra of the solid samples of compounds PP1, PP2 (**b**).

Cyclic voltammetry (CV) measurements were performed for the dilute dichloromethane solutions of compounds PP1 and PP2 in order to experimentally determine energy levels, which are important for the evaluation of the applicability of the compounds in electronic devices. The voltammograms of compounds PP1 and PP2 are presented in Figure 4a. The obtained data are presented in Table 2.

The ionization potential values of the dichloromethane solutions of the compounds were estimated from the oxidation potential values obtained from the cyclic voltammograms. The influence of methoxy group introduction to PP2 is evident as it results in smaller ionization potential *IP^CV^* (Table 2). Electron affinity *EA^CV^* values of dichloromethane solutions of PP1 and PP2 are relatively similar as their acceptor moiety is the same pyrimidine.

The ionization potentials *IP^EP^* of the solid samples of PP1 and PP2 were recorded by the electron photoemission spectrometry in air (Table 2, Figure 4b). The IP values are found to be comparable and in the same trend. The theoretically determined highest occupied molecular orbital (HOMO) values are also in agreement with the experimental results.

### 3.3. Photophysical Properties

**Figure 5:**
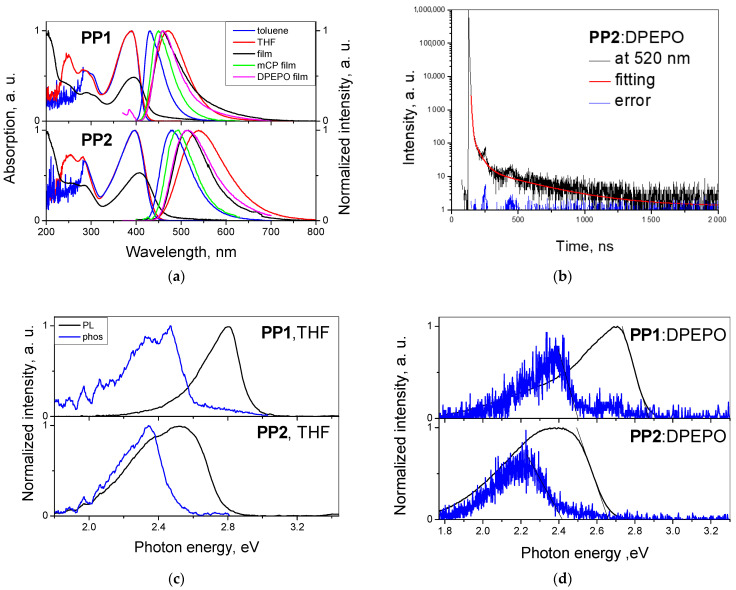
Absorption and PL spectra of dilute toluene and THF solutions, neat films, and films of the compounds doped in mCP and DPEPO (20 wt.%) (**a**) at room temperature. PL decay curve of the film of PP2 doped in DPEPO (20 wt.%) recorded in vacuum at 300 K (**b**). PL and phosphorescence spectra of the THF solutions of PP1 and PP2 (**c**) and the films of the compounds doped in DPEPO (20 wt.%) (**d**) recorded at 77 K.

To gain a deeper understanding of the characteristics of the absorption bands exhibited by the target compounds, TD-DFT calculations were performed (Figure S10). The results of photophysical investigation of compounds of PP1 and PP2 are collected in Table 3, Figure 5. Absorption spectra of the solutions of PP1 and PP2 showed peaks of transitions towards various excited states (Figure 5a). The bands at ca. 240 can be attributed to π-π* transitions of the aromatic cycles [37,38,39,40]. The peaks at ca. 300 and ca. 390 nm can be assigned to the π-π* transitions on the phenylamino groups and intramolecular charge transfer (ICT) bands of absorption.

The theoretical UV spectra of both compounds have several dominating transitions resulting in two absorption bands (Figure S10). The lowest energy bands (LEB) of both UV spectra correspond to the HOMO → LUMO transition. This transition is a mixture of local and ICT transitions, originating towards the whole molecule. The absorption band at ca. 290 nm of compound PP1 is mainly characterized by two transitions. The first excitation with an oscillator strength of 0.275 corresponds to the HOMO → LUMO + 6 transition. This transition is from the extended orbitals on the donor fragments and phenyl moiety towards the diphenylamine fragments. It can be ascribed as a mixture of LE and ICT. The second dominating transition with the oscillator strength of 0.3202 corresponds to the HOMO-2 → LUMO + 1 transition. This transition can be ascribed as LE and is localized on the electron-accepting moieties. The absorption band at ca. 290 nm of compound PP2 also reflects two dominating transitions, whose origin is the same as for compound PP1.

The attachment of methoxy substituents to diphenylamino groups results in the bathochromic shift of the lowest energy band of absorption by ca. 10 nm and emission spectral band by ca. 50–70 nm. The positive solvatochromism is confirmed by the continuous bathochromic shifts of the PL peaks upon increasing the solvent polarity from non-polar toluene to polar THF. Thus, PL attributed to the ICT is shifted from dark blue to blue for PP1 and from blue to yellowish green for PP2. Enhanced intermolecular interactions in the solid state cause the substantial bathochromic shifts of both absorption and PL peaks and the appearance of low energy absorption bands in the UV spectra of both compounds in comparison to the corresponding spectra of the solutions of the compounds. The PL peaks of the films of the compounds are closer to the respective peaks of their THF solutions rather than to the toluene solutions. It is a manifestation of the noticeable self-polarity of the film. For the investigation of the photophysical properties of the films of the molecular dispersions of PP1 and PP2 in electronically active materials 1,3-di(carbazol-9-yl)benzene (mCP) (*E_T1_* = 2.9 eV; *ε* = 2.84) and bis[2-(diphenylphosphino)phenyl] ether oxide (DPEPO) (*E_T1_* = 3.1 eV; *ε* = 6.12) [41] were selected as host matrixes. The doping concentration was 20 wt.%. PL peaks of the films of the compounds doped in DPEPO are redshifted compared to the PL peaks of films of the compounds doped in less polar mCP, highlighting the ICT origin of the emission and the effect of the polarity of the media, i.e., of the host. The location of PL peaks is almost identical for the neat films and the films of the compounds doped in DPEPO manifesting the relatively high inherent polarity of PP1 and PP2 with *ε* close to that of DPEPO. PL quantum yield (PLQY) values of toluene solutions of PP1 and PP2 are 80 and 63%, respectively (Table 3). PP2 exhibits lower PLQY than PP1 in all kinds of samples as the methoxy attachments stimulate free vibrations/rotations due to flexible linkages between the pyrimidine core and the donor moieties. The methoxy groups also enhance intermolecular interactions in the solid state causing a further decrease in the efficiency of PL in the solid state of PP2 to a greater extent than of PP1 (Table 2). The PLQY values of the films of the compounds doped in mCP are higher than those of the corresponding DPEPO-based films due to the higher polarity of mCP. The solid-state samples exhibit less efficient emission than the toluene solutions highlighting aggregation-caused quenching. The films of guest–hosts systems of both the compounds exhibit higher PLQY than the films of neat compounds proving the efficient electronic excitation energy transfer from the host matrix to the emitter.

PL decay curves of the neat layers and of the layers of guest–host systems recorded at room temperature in the air indicate that the emission is prompt fluorescent with lifetimes shorter than 10 ns. Dilute toluene solutions of PP1 and PP2 exhibit an increase in PL intensity by factors of 1.16 and 1.36, respectively, upon deoxygenation (Figure S11). This is a manifestation of triplet exciton utilization in emission, which is quenched in the presence of oxygen due to the electronic excitation being externally converted through collisional interactions with molecular oxygen [42]. For this reason, the PLQY of the films of the compounds can be increased if the oxygen-free environment is ensured. The spectral profile remains unchanged after deoxygenation, highlighting the absence of room-temperature phosphorescence. The films of compounds doped in DPEPO (20 wt.%) exhibit a long lifetime of emission of up to 0.4 μs (Figure 5b and Figure S12). The low-energy shoulder of the PL spectrum of the molecular mixture of PP1 and DPEPO is evident. The subdivision of the band of the PL spectrum of the film of a molecular mixture of PP2 and DPEPO is also possible as the band is broader than that of host-free films. The normalized PL spectra of the films of the molecular mixtures of the compounds with DPEPO exhibit a slight enhancement of the emission after removing air. This observation is attributed to the triplet energy harvesting (Figure S13). Investigation of the photophysical properties of the compounds at the different temperatures showed no indications of thermal activation of the long-lifetime emission excluding TADF as a cause of the emission of both guest–DPEPO systems (Figure S14). PL and phosphorescence spectra of THF solutions were recorded at the liquid nitrogen temperature (Figure 5c). The values of energy of the first singlet *E_S1_* and triplet *E_T1_* excited states were estimated from the onsets of the fluorescence and phosphorescence spectral bands, respectively. The solutions are characterized by relatively high singlet–triplet energy splitting (Δ*E_ST_*). The Δ*E_ST_* values of the films of the molecular mixtures of PP1 and PP2 with DPEPO are also too big for efficient TADF (Figure 5d). The photophysical characteristics of THF solutions of the compounds and the films of their molecular mixtures with DPEPO recorded at 77 K correlate to a great extent. The occurrence of room temperature phosphorescence is unlikely due to the small, detected lifetime values and the fact that the spectral profiles do not change upon the increase in temperature (Figure S14). The detected singlet and triplet ICT bands correspond to several electronic transitions accompanying the upconversion of triplet excitons with higher energy levels than T_1_ resulting in delayed fluorescence (DF). Electronic excitation energy transfer from host to guest occurs in a manner that stimulates RISC, as the set of energy levels of excited states is tuned by the polarity of the host and dipole–dipole interactions.

**Figure 6:**
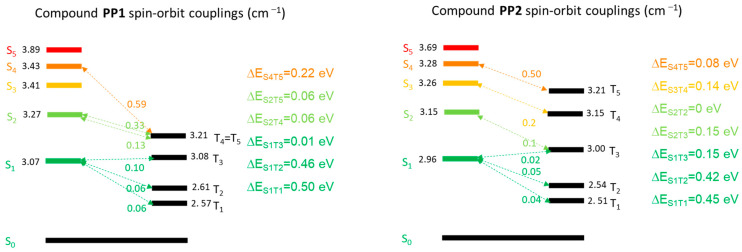
Calculated spin–orbit coupling values of compounds PP1 and PP2.

The theoretical calculations supported the experimental observations. The calculated values of singlet–triplet energy values of both compounds are also quite high (Figure 3, Figure 5d and Figure 6). The calculated spin–orbit coupling matrix element (SOCME) between the first five singlet and triplet states has revealed a potential hot exciton emission pathway of the excited states (Figure 6). The S_2-4_–T_2-5_ gaps are lower than 0.22 eV and result in efficient ISC from the upper states. This is also substantiated by relatively higher SOCME values of the upper triplet states with S1 in comparison to the lowest excited states (S_1_-T_1_). This allows several RISC channels to emerge from high-lying states, thus enabling a hot exciton emission mechanism. Such a mechanism is advantageous, as in the case of compounds PP1 and PP2, the S_1_ state is characterized by LE and ICT. In this case, the LE emission is dominant when compared to the ICT contribution to the S_1_ state emission resulting in high PLQY.

### 3.4. Charge-Transporting Properties

**Figure 7:**
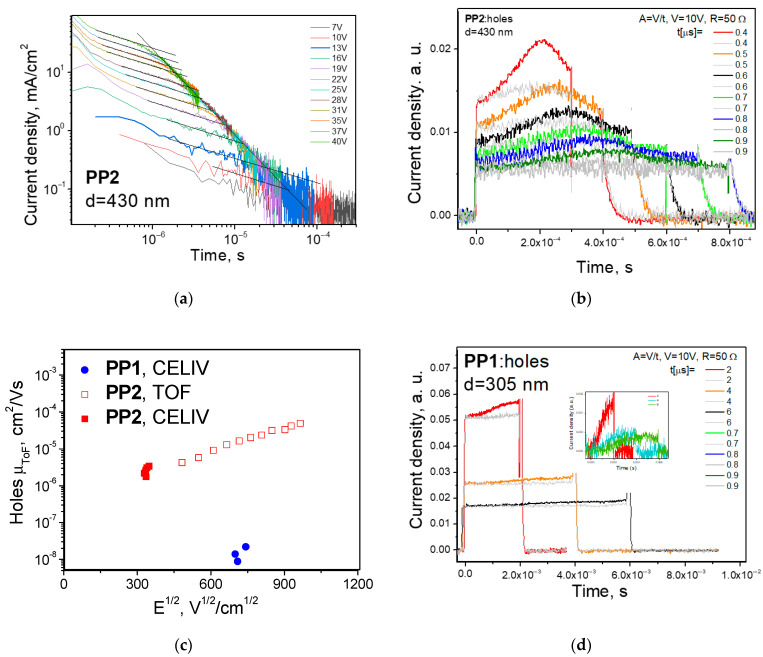
TOF (**a**), dark-CELIV (grey), and photo-CELIV (colored) (**b**,**d**) signals. Inset shows signals which are subtraction of dark-CELIV from photo-CELIV. Hole mobility plots as the Poole–Frenkel function of electric fields (**c**) (*µ = µ_0_* ×*exp(α × E^1/2^*), where µ_0_ and α refer to zero-field mobility and field dependence, respectively (**d**) [44]).

The measurements of charge carrier mobilities were carried out using the time-of-flight (TOF) and charge extraction by linearly increasing voltage (CELIV) methods [43]. The same vacuum-deposited films of PP1 and PP2 were used for the measurements by both techniques. The samples with the structure ITO/film/Al with an active area of 6 mm^2^ were used. The thicknesses (d) of the layers varied from 305 to 430 nm. For the sample of PP2, the hole photocurrent transients were recorded and plotted using log–log scales at various external electric fields when positive voltages (V) were applied to ITO (Figure 7a). The TOF signals are distinguished by a plateau and a tail, which are identified by auxiliary lines. The shapes of the TOF signals obtained indicate low dispersity of charge transport. To determine hole mobilities by the formula *µ = d*^2^*/V × t_tr_* under the different electric fields, values of transit times *t_tr_* were taken at the intersection of auxiliary lines. The hole mobility value of 4.9 × 10^−5^ cm^2^/V × s was obtained for PP2 at an electric field of 9.2 × 10^5^ V/cm by TOF measurements. The CELIV measurements were also performed for PP2, to estimate hole mobilities by the formula *µ* = 2*d*^2^*/A* × *t_max_*^2^. The times *t_max_* were taken at the maximum current density of photo-CELIV signals seen as the peaks in Figure 7b. The dark-CELIV and photo-CELIV signals were collected without and under laser excitation (355 nm), respectively. In a CELIV experiment, *A = U/t_pulse_* refers to the slope of the triangle voltage pulse that is applied to the sample [43]. The CELIV measurements of PP2 yielded lower hole mobility values (e.g., 3.4 × 10^−6^ cm^2^/V × s at an electric field of ca. 1.2 × 10^5^ V/cm) than TOF due to the differences in electric fields, but they were consistent with the results of TOF measurements (Figure 7c). It was not possible to determine *t_tr_* values for PP1 due to strongly dispersive charge transport. However, *t_max_* values were obtained for PP1 since CELV measurements are less sensitive to charge transport dispersity (Figure 7d). The hole mobility of PP1 was found to be 2.2 × 10^−8^ cm^2^/V × s at the electric field of approximately 5.5 × 10^5^ V/cm. The higher hole mobility of PP2 compared with that of PP1 highlights the beneficial impact of methoxy units on the hole transport.

### 3.5. Electroluminescent Properties

**Figure 8:**
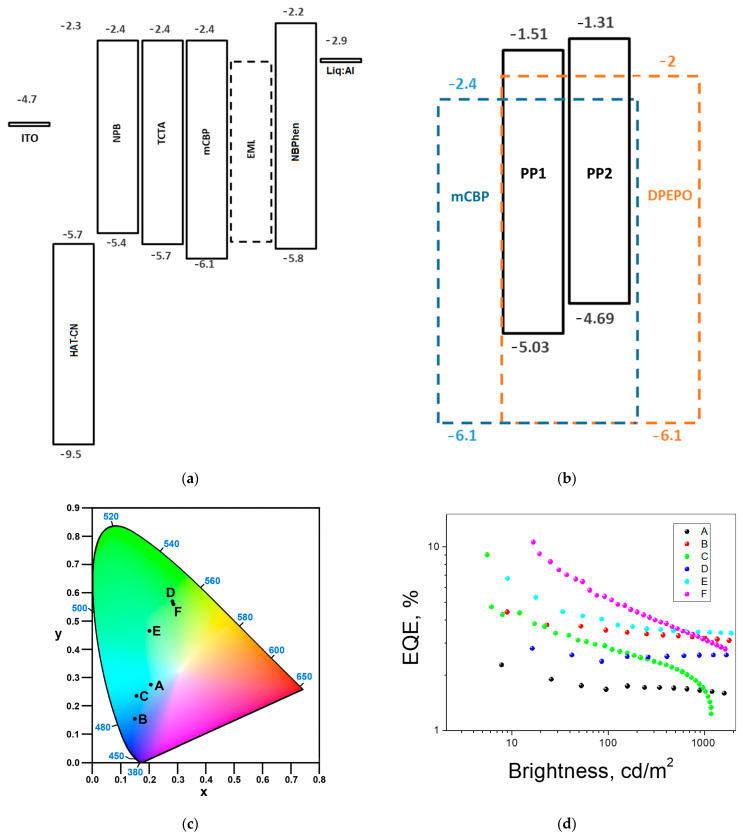

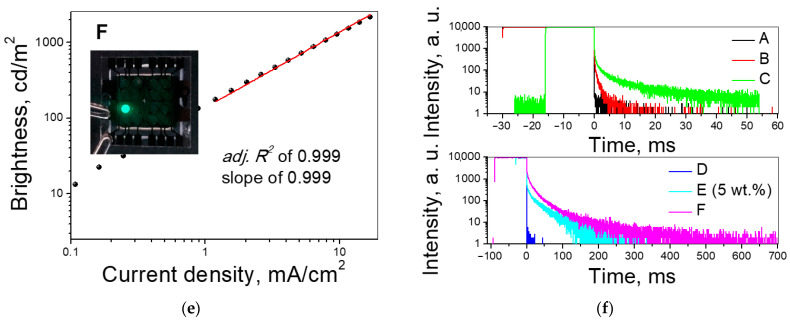
OLED equilibrium energy level diagram (**a**); EML energy level diagram (**b**); 1931 CIE diagram, inset: photograph of OLED C (**c**); correlation of EQE and brightness (**d**); correlation of brightness and current density for F OLED, inset: photograph of OLED F (**e**); and TREL decay curves (**f**).

Compounds PP1 and PP2 were tested as emitters of OLEDs using device structure: ITO/HAT-CN (5 nm)/NPB (40 nm)/TCTA (10 nm)/mCBP (10 nm)/EML (50 nm)/NBPhen (30 nm)/Liq/Al. 1,4,5,8,9,11-Hexaazatriphenylenehexacarbonitrile (HAT-CN) and Liq were used for the deposition of hole and electron injection layers, respectively. N,N′-Di(1-naphthyl)-N,N′-diphenyl-(1,1′-biphenyl)-4,4′-diamine (NPB) and tris(4-carbazoyl-9-ylphenyl)amine (TCTA) were utilized for the deposition of hole transporting layers. The layer of 3,3′-di(9H-carbazol-9-yl)-1,1′-biphenyl (mCBP) was deposited for the exciton blocking. 2,9-Dinaphthalen-2-yl-4,7-diphenyl-1,10-phenanthroline (NBphen) was employed for the deposition of the electron transporting layer. The diagram of the structure of the device is presented in Figure 8a. The emitting layer (EML) was designed to be a doping-free layer, or a layer of the emitter doped in mCBP or DPEPO for devices A-F (Table 4). The diagram of energy levels of the materials used for the deposition of EML is given in Figure 8b.

EL spectra and characteristics of OLEDs are presented in Figures S15–S20. The EL spectra correlate to a great extent with the PL spectra of the compounds. However, doping of the emitters into mCP significantly blueshifts the emission peak due to guest–host interactions and the polarity effect. The 1931 Commission Internationale de l’éclairage (CIE) colour space diagram coordinates recorded at 1000 cd/m^2^ of OLEDs are shown in Figure 8c (Table 4). Similarly to the PL spectra of the films of the molecular mixtures of PP1 and DPEPO, the EL spectra of OLED C possessing the respective EML have a double peak signature where the relation between the intensity of the two peaks changes upon increasing the voltage. The utilization of the guest–host systems resulted in providing electronic excitation energy of the emissive species in a way that stimulated the overall efficiency of OLEDs. The LUMO energy level of DPEPO is better aligned to the LUMO levels of PP1 and PP2 than that of mCP. This is partially the reason for the better efficiencies of OLEDs C and F compared to those of devices B and E. Nevertheless, it is worth noting that these devices also broke the theoretical limit of maximum external quantum efficiency (EQE) of ca. 5% for devices without advanced out-coupling and substrates based on prompt fluorescent emitters (Table 4). The data point to the efficient utilization of triplet excitons in the DF with the maximum EQE of 10.6% observed for OLED with EML consisting of PP2 and DPEPO (Figure 8d). PP2 exhibited better performance than PP1 in both host-containing and host-free OLEDs. This observation can be attributed to the dispersive charge transport of PP1 that suppresses the quantum efficiency and deteriorates the charge balance on the recombination sites (Figure 7).

The application in OLEDs of the guest–host systems provided an opportunity to check the probability of occurrence of the phenomenon of triplet–triplet annihilation (TTA) in the studied EML. Since TTA is based on the annihilation of two triplet excitons to form one singlet exciton with the energy twice higher, the intensity of emission originating from TTA has a quadratic dependence on the initial population of triplet excitons [45,46,47]. As the current density is increased, the initial triplet energy is drastically increased, changing the kinetics of physical processes happening during EL. Consequently, the mentioned quadratic dependence becomes linear at bigger values of initial triplet populations [45,46,47]. That dependence corresponds to the slope of two of the linear fit of the correlation of brightness and current density, which switches to the slope of one at higher current densities [45,46]. The expected range of the switch between the slopes is reportedly varied in the diapason of ca. 1–30 mA/cm^2^ [44,45]. The linear fit of the dependence of brightness and current density derived from the characterization of OLEDs utilizing guest–host systems that were close to breaking or broke the 5% limit of maximum EQE was completed (Figure 8e and Figure S21). The fitting was completed mostly in the range of 1–10 mA/cm^2^. The values of the slopes vary from 0.88 to 0.999 with the *adj. R*^2^ being of ca. 0.999. This undoubtfully excludes TTA as a reason for high maximum EQE.

To further study the nature of EL, transient EL (TREL) profiles of the OLEDs were recorded (Figure 8f and Figure S22) at the wavelengths of the corresponding EL spectral peaks. The TREL signal is obtained by the measurement of the EL intensity of OLED in a time-resolved manner after a constant voltage pulse of 6 V. When the operational voltage is applied, charge carriers are collected at the recombination sites. The following decay of the intensity of EL represents the decrease in exciton population after switching off the voltage. The so-called rise time is the time needed to reach the saturation of EL intensity. It is a characteristic of the hole and electron drift mobility as a manifestation of the exciton formation upon applying a constant voltage [48]. In our case, the rise time is negligible, and the saturated signal is remarkably stable, having no spikes exhibiting the stability of the EL, no charging and discharging [49], and the absence of the appearance of phenomena affecting the exciton population due to the storage of triplet excitons with long lifetimes inducing additional exciton population. The multiexponential fit of the TREL curves provides the values of a lifetime of the exciton populations related to the DF. OLEDs A and D with the doping-free layers of PP1 and PP2, respectively, exhibit practically no DF. The TREL signal is stronger for the devices with EML utilizing hosts mCBP and DPEPO, which are more efficient than the fabricated OLED. This manifests the direct contribution of triplet excitons in the increase in EQE. The lifetime values of the exciton population related to the DF are collected in Table S1. The presence of several components of the decay curves is caused by phenomena such as exciton–polaron and exciton–exciton annihilation processes [50] enhanced by the increased exciton density at interfaces of the layers during saturated EL intensity. While TTA is expected in such circumstances, it was shown previously (Figure 8e and Figure S22) that TTA is not prominent and has no significant contribution to the efficiency of the OLEDs at least at low current densities. The lifetimes of the exciton population of the components of the TREL signals reach the values of 37 for device C based on the EML consisting of PP1 and DPEPO and 76 ms for device F with EML consisting of PP2 and DPEPO. These data highlight the enhancement of RISC due to the favorable host–guest energy transfer aligning with the results of the PL measurements. The triplet exciton emission results in the maximum EQE of OLEDs reaching 10.6%.

## 4. Conclusions

The derivatives of 2-phenylpyrimidine containing triphenylamino or 4,4′-dimethoxytriphenylamino donor moieties were synthesized and their thermal, electrochemical, photophysical, charge-transporting as well as electroluminescent properties were studied. Attachment of methoxy groups to diphenyl amino moieties resulted in changes in the emission of the solid films from blue peaking at 460 nm to green–blue peaking at 513 nm. It is shown that the attachment of methoxy groups resulted in a significant increase in hole drift mobility up to 4.9 × 10^−5^ cm^2^/V × s at an electric field of 9.2 × 10^5^ V/cm. The compound having no methoxy groups exhibited more efficient emission and better performance in OLEDs. The devices with both a neat emitting layer and guest–host systems based on the emitter with methoxy groups exhibited higher efficiency than organic light-emitting diodes based on an emitter having no methoxy groups. This observation is attributed to the better charge balance in the layers of 4,6-bis((4-di(4-metoxyphenil)amino)-phenil)-2-phenilpyrimidine. The electroluminescent properties of the compounds are consistent with their photophysical properties, showing that the delayed fluorescence occurs only after doping the emitters in hosts. This leads to the breaking of the limit of 5% of external quantum efficiency of OLEDs based on prompt fluorescence. The transient electroluminescence data confirm the long-lived emission of the guest–host emitting layer. It is demonstrated that bis[2-(diphenylphosphino)phenyl]ether oxide (DPEPO) is a superior host relative to 3,3′-di(9H-carbazol-9-yl) for stimulating delayed fluorescence. Thermal activation of delayed fluorescence was not detected, excluding the thermally activated delayed fluorescence as a cause of long-lived emission. Based on the analysis of electroluminescent properties of the compounds, it also concluded that triplet–triplet annihilation is also not the reason for efficient triplet exciton utilization leading to the maximum external quantum efficiency of devices of up to 10.6%. As the spectral profiles of the films of the compounds doped in DPEPO remain unchanged after the removal of oxygen the upper reverse intersystem crossing according to the hot exciton mechanism is postulated. The hypothesis is supported by the detection of the spectral contributions of locally excited and intramolecular charge transfer states into emission.

## Figures and Tables

**Table 1:** Thermal characteristics of compounds PP1, PP2.

Compound	*T_d_*, °C	*T_m_*, °C	*T_g_*, °C	*T_cr_*, °C
PP1	397	249	106	221
PP2	438	213	103	-

**Table 2:** Electrochemical characteristics of PP1 and PP2.

	*E^ox^ _½_* vs. *_FC_*, V	*IP^CV^*, eV	*IP^EP^*, eV	*EA^CV^*, eV	*E_g_^theo^*, eV
PP1	0.48	5.28	5.82	2.33	3.52
PP2	0.28	5.08	5.63	2.26	3.40

*IP^CV^*—ionization potential calculated by the formula *I_P_^CV^* = 4.8 + *E^ox^_½_* vs. *_FC_*; *IP^EP^*—ionization potential determined by photoemission; *E^ox^*_½_ vs. *_FC_*—oxidation potentials versus ferrocene; *EA^CV^*—electron affinity calculated by the formula *EA^CV^ = IP^CV^ − E_g_^opt^* (toluene); *E_g_^theo^*—HOMO-LUMO gap estimated using theoretical values.

**Table 3:** Photophysical properties of PP1, PP2.

Compound	Sample	*λ^UV^_max_*, nm	*E_g_^opt^*, eV	*λ^FL^_max_*, nm	Stokes Shift, nm	Lifetime, ns	PLQY, %	*E_S1_*, eV	*E_T1_*, eV	Δ*E_st_*, eV
PP1	Toluene	-/390	2.95	431	41	-	80	-	-	-
	THF	284/390	2.91	470	80	-	-	2.93	2.61	0.32
	Film	396	3.13	460	64	1.46/8.20	32	-	-	-
	20 wt.% mCP film	-	-	450		2.94/9.28	62	-	-	-
	20 wt.% DPEPO film	-	-	466		2.56/6.86 DF: 218.86 (at 460 nm), 381.59 (at 530 nm)	41	2.87	2.5	0.37
PP2	Toluene	-/397	2.82	479	82	-	63	-	-	-
	THF	281/397	2.79	539	142	-	-	2.77	2.49	0.28
	Film	407	2.51	513	106	2.08/6.16	23	-	-	-
	20 wt.% mCP film	-	-	494		3.41/7.14	34	-	-	-
	20 wt.% DPEPO film	-	-	509		3.65/7.89 DF: 372.21 (at 521 nm)	26	2.66	2.42	0.24

**Table 4:** EL characteristics of OLEDs.

OLED	EML	CE_max_,cd/A	PE_max_, lm/W	EQE_max_, %	1931 CIE_x,y_
A	PP1	3.5	3.0	2.3	(0.21, 0.27)
B	PP1:mCBP	5.2	3.5	4.4	(0.15, 0.15)
C	PP1:DPEPO	11.4	7.3	9.0	(0.15, 0.23)
D	PP2	8.2	5.7	2.8	(0.28, 0.57)
E	PP2:mCBP	13.1	7.9	6.7	(0.20, 0.46)
F	PP2:DPEPO	27.6	11.4	10.6	(0.28, 0.56)

## Data Availability

The data presented in this study are available on request from the corresponding author.

## Supplementary Materials

The following supporting information can be downloaded at: https://www.mdpi.com/article/10.3390/ma17061357/s1, Figure S1: ^1^H NMR spectrum of 4,6-bis(4-chlorophenyl)-2-phenylpyrimidine in CDCl_3_; Figure S2: ^13^C NMR spectrum of 4,6-bis(4-chlorophenyl)-2-phenylpyrimidine in CDCl_3_; Figure S3: Mass spectrum of 4,6-bis(4-chlorophenyl)-2-phenylpyrimidine; Figure S4: ^1^H NMR spectrum of PP1 in CDCl_3_; Figure S5: ^13^C NMR spectrum of PP1 in CDCl_3_; Figure S6: Mass spectrum of PP1; Figure S7. 1H NMR spectrum of PP2 in CDCl3; Figure S8. ^13^C NMR spectrum of PP2 in CDCl_3_; Figure S9: Mass spectrum of PP2. Figure S10: Theoretical UV spectra (dielectric constant evaluated ε = 6), and excited state geometries of compounds PP1 (a) and PP2 (b). (Grey colour: carbon; blue: nitrogen; red: oxygen; white: hydrogen); Figure S11: PL spectra of dilute toluene solutions of PP1 and PP2 after deoxygenation; Figure S12: PL spectra of the films of PP1 and PP2 doped in DPEPO (20 wt.%) in vacuum at 300 K; Figure S13: PL spectra of the films of PP1 and PP2 doped in DPEPO (20 wt.%) before and after removal of air; Figure S14: PL spectra of the films of PP1 and PP2 doped in DPEPO (20 wt.%) in inert atmosphere at different temperatures; Figure S15: OLED A: EL spectra; luminescence and current density vs. voltage correlations; current and power efficiency, EQE vs. luminescence correlations; Figure S16: OLED B: EL spectra; luminescence and current density vs. voltage correlations; current and power efficiency, EQE vs. luminescence correlations; Figure S17: OLED C: EL spectra; luminescence and current density vs. voltage correlations; current and power efficiency, EQE vs. luminescence correlations; Figure S18: OLED D: EL spectra; luminescence and current density vs. voltage correlations; current and power efficiency, EQE vs. luminescence correlations; Figure S19: OLED E: EL spectra; luminescence and current density vs. voltage correlations; current and power efficiency, EQE vs. luminescence correlations; Figure S20: OLED F: EL spectra; luminescence and current density vs. voltage correlations; current and power efficiency, EQE vs. luminescence correlations; Figure S21. Correlation of brightness and current density for OLEDs B, C, E and F; Table S1: Lifetime values of the exciton population derived from TREL decay curves of OLEDs related to the DF; Figure S22: TREL decay curves of OLEDs. Refs. [51,52] are cited in the Supporting Information.
