# Supplementary material for: Derivatives of Phenyl Pyrimidine and of the Different Donor Moieties as Emitters for OLEDs
Source: Materials (Basel). 2024 Mar 15;17(6):1357. doi: 10.3390/ma17061357 (PMC10972299; doi:10.3390/ma17061357)
Supplement: Supplementary file 1 [file materials-17-01357-s001.zip › materials-2885635-supplementary.pdf]

Supplementary Materials

Derivatives of Phenyl Pyrimidine and of the Different Donor Moieties as Emitters for OLEDs

Hryhorii Starykov [1], Oleksandr Bezvikonnyi [1,2], Karolis Leitonas [1], Jurate Simokaitiene [1], Dmytro Volyniuk [1], Eigirdas Skuodis [1], Rasa Keruckiene [1] and Juozas V. Grazulevicius [1*]

[1] Department of Polymer Chemistry and Technology, Faculty of Chemical Technology, Kaunas University of Technology, K. Barsausko st. 59, LT-51423 Kaunas, Lithuania
[2] Department of Physics, Faculty of Mathematics and Natural Sciences, Kaunas University of Technology, Studentų st. 50, LT-51369 Kaunas, Lithuania
* Correspondence: juozas.grazulevicius@ktu.lt

The geometry and electronic structure of compounds PP1–2 in ground and excited states were investigated within density functional theory using B3LYP functional with 6-31++G(d) basis set. Energy gap between the singlet and triplet states (ΔE_{S-T}) was calculated as energy difference between S1 and T1 excited states having the ground state geometry with dielectric constant value of ε=6. All of the calculations were carried out using Gaussian16 program[51]. SOCME values were calculated using software ORCA[52].

Photohysical measurements were done via Avantes AvaSpec-2048XL and Edinburgh Instruments FLS980 spectrometers. PLQY were estimated by the absolute method using the integrating sphere and FLS980 with the Xe lamp as an excitation source at 330 nm. Decay curves were measured using FLS980 and PicoQuant LDH-D-C-375 laser (374 nm of the excitation wavelength) and the microsecond lamp. Deoxygenation of the solutions were made by the argon bubbling.

The PE spectroscopy setup consisted of a CM110 1/8m monochromator, 6517B Keithley electrometer and a deep ultra-violet ASBN-D130-CM deuterium lamp. Keithley 6517B electrometer and EKSPLA NL300 laser (355 nm of excitation wavelength) were used for the time-of-flight (TOF) method. Photocurrent transients were recorded via Tektronix TDS 3032C oscilloscope. The samples for PE and TOF were made using the thermal vacuum deposition via Kurt J. Lesker equipment in-built in an MB EcoVap4G glove box.

ITO substrates for fabrication of OLEDs were treated by a UV ozone cleaner for 10 min. The deposition of layer by the thermal vacuum deposition technique was done utilizing Oerlikon-Leybold UNIVEX 350G evaporator system in-built into a glove-box. SQC 310 controller with two quartz crystals sensors was used for the control of deposition rate (0.5-1 Å/sec). OLEDs were encapsulated by glass caps via UV curable epoxy. The electroluminescent and current density – voltage characteristics were measured simultaneously using the McScience M7000 Auto I-V-L Test System. The EL data were carried out at room temperature under ambient atmosphere.

TREL signals were measured out by applying a rectangular voltage pulse using the Arbitrary/Function Generators Tektronix AFG3000C to the OLEDs. Synchronization of Tektronix pulse generator and FLS980, photomultipliers were used to analyse collected EL. FLS980 software was used for the estimation of 1931 CIE coordinates.

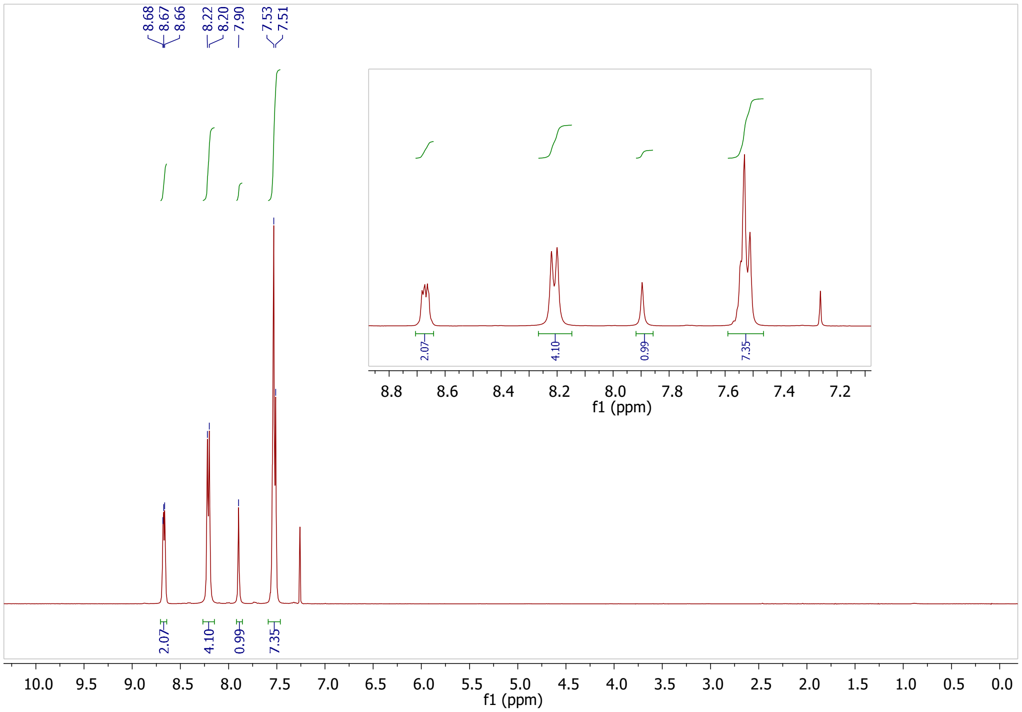

Figure S1. ^{1}H NMR spectrum of 4,6-bis(4-chlorophenyl)-2-phenylpyrimidine in $CDCl_3$.

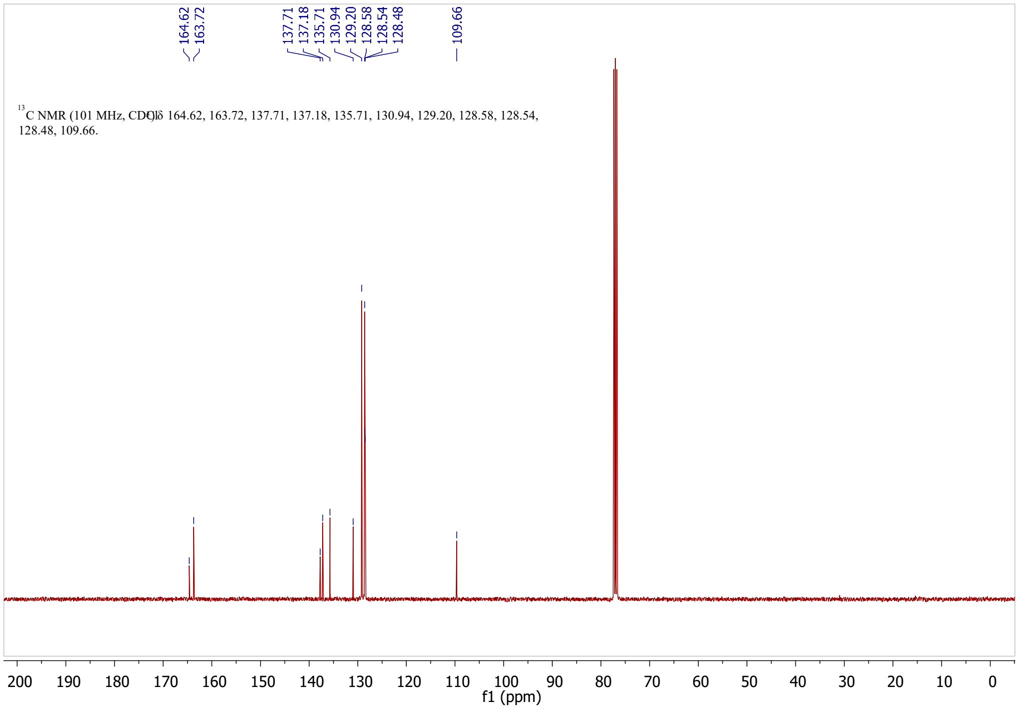

Figure S2. ^{13}C NMR spectrum of 4,6-bis(4-chlorophenyl)-2-phenylpyrimidine in $CDCl_3$.

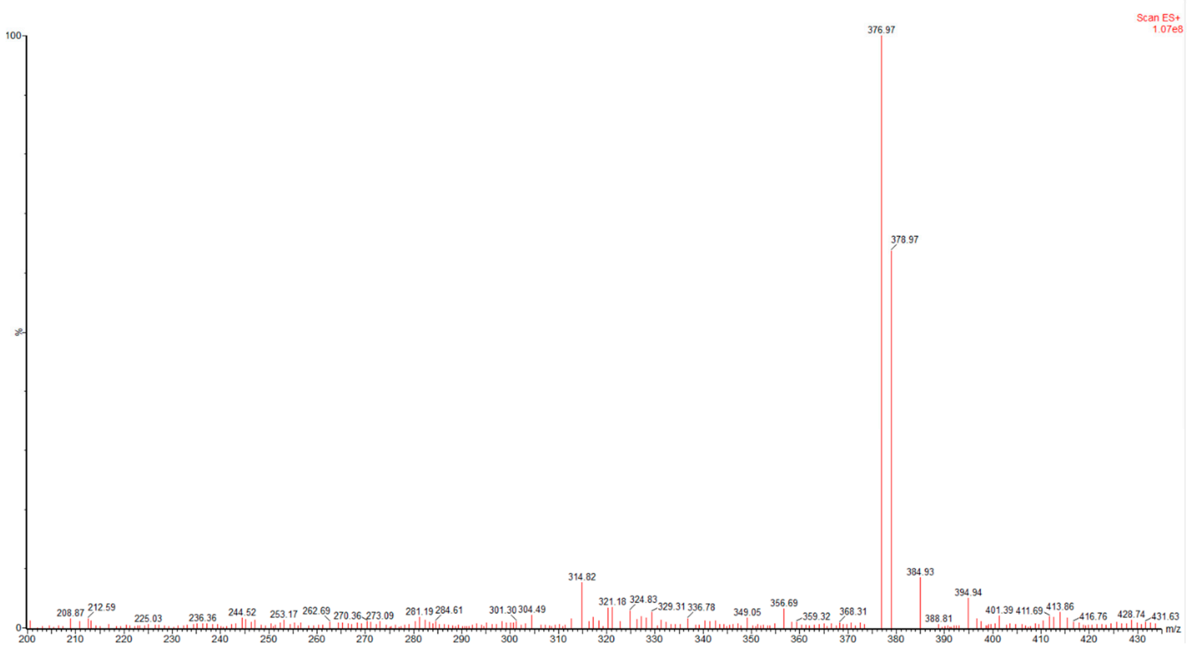

Figure S3. Mass spectrum of 4,6-bis(4-chlorophenyl)-2-phenylpyrimidine.

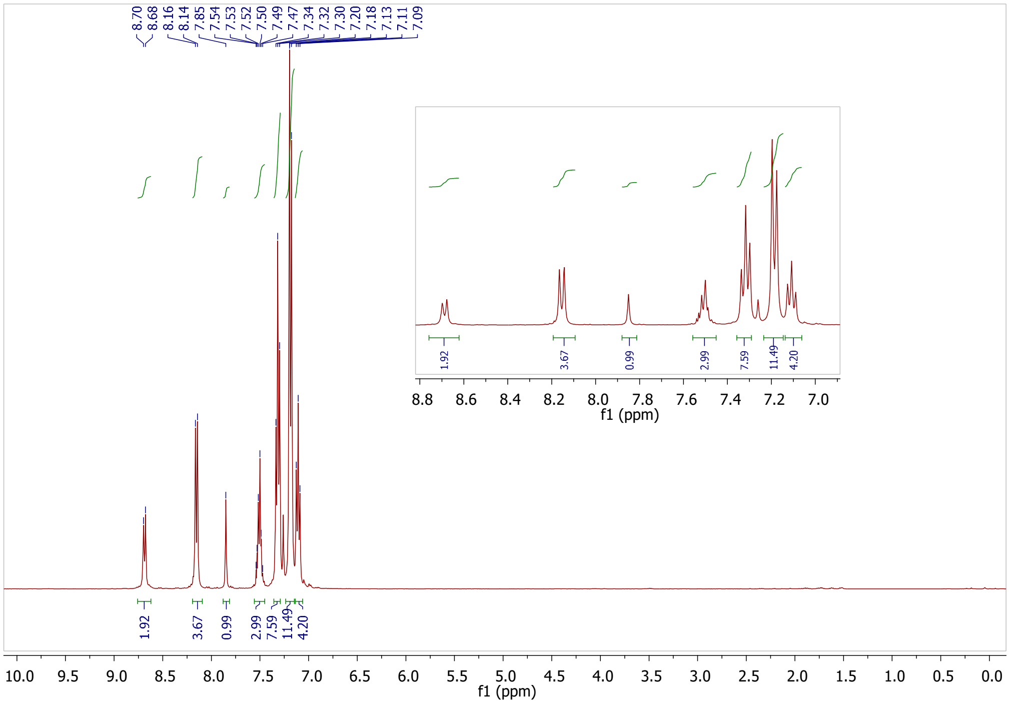

Figure S4. ^{1}H NMR spectrum of PP1 in $CDCl_3$.

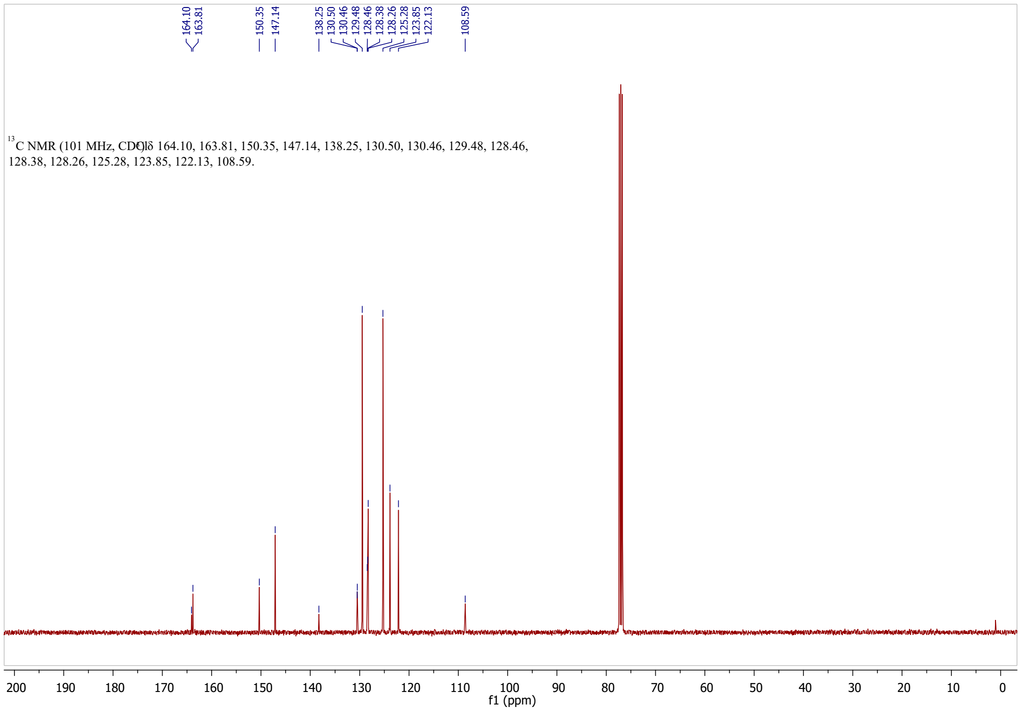

Figure S5. ^{13}C NMR spectrum of PP1 in $CDCl_3$.

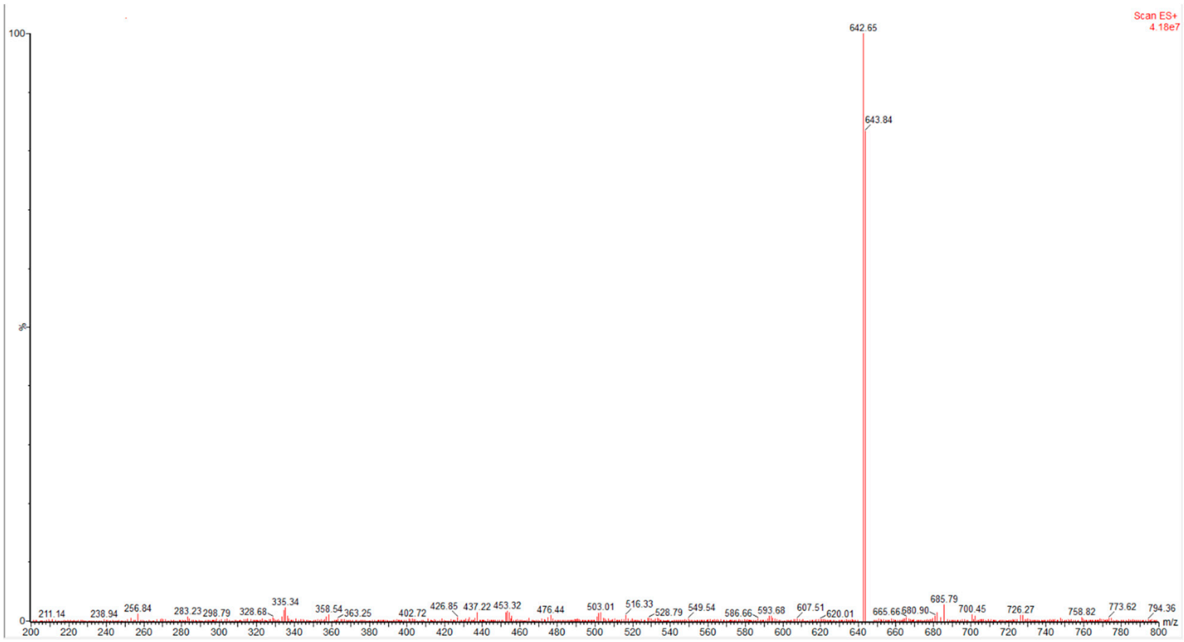

Figure S6. Mass spectrum of PP1.

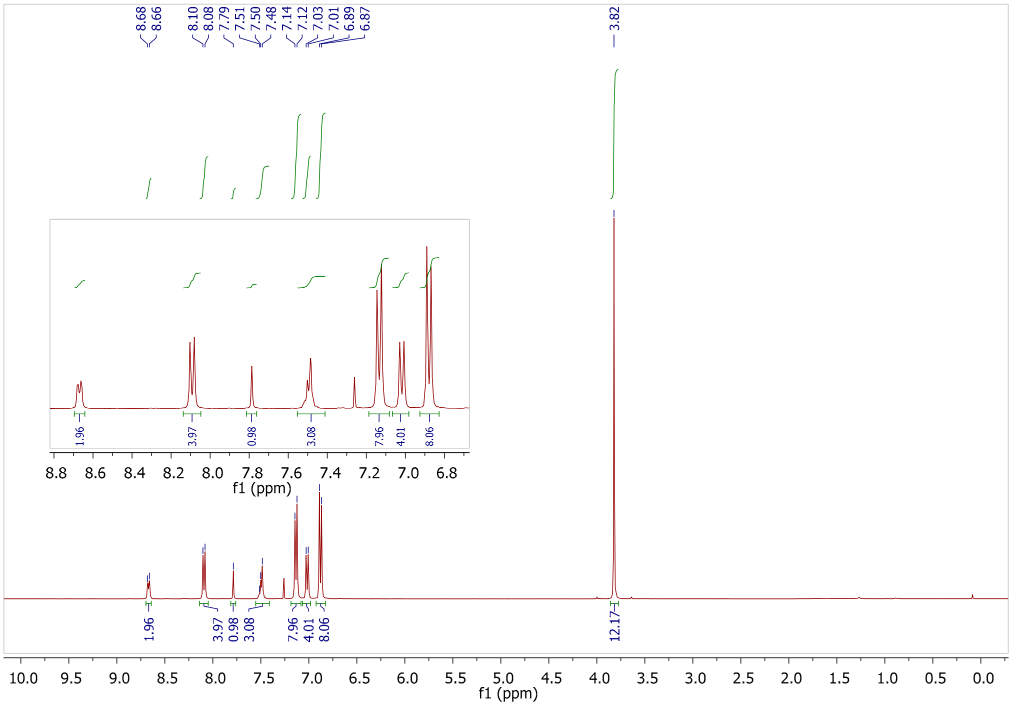

Figure S7. ^{1}H NMR spectrum of PP2 in $CDCl_3$.

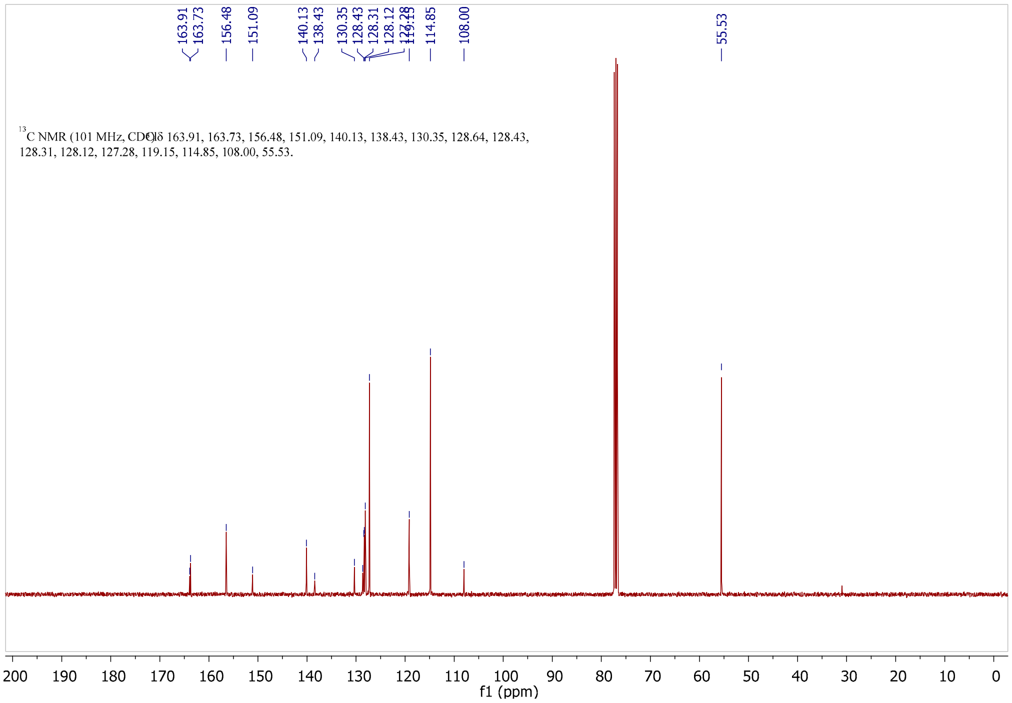

Figure S8. ^{13}C NMR spectrum of PP2 in $CDCl_3$.

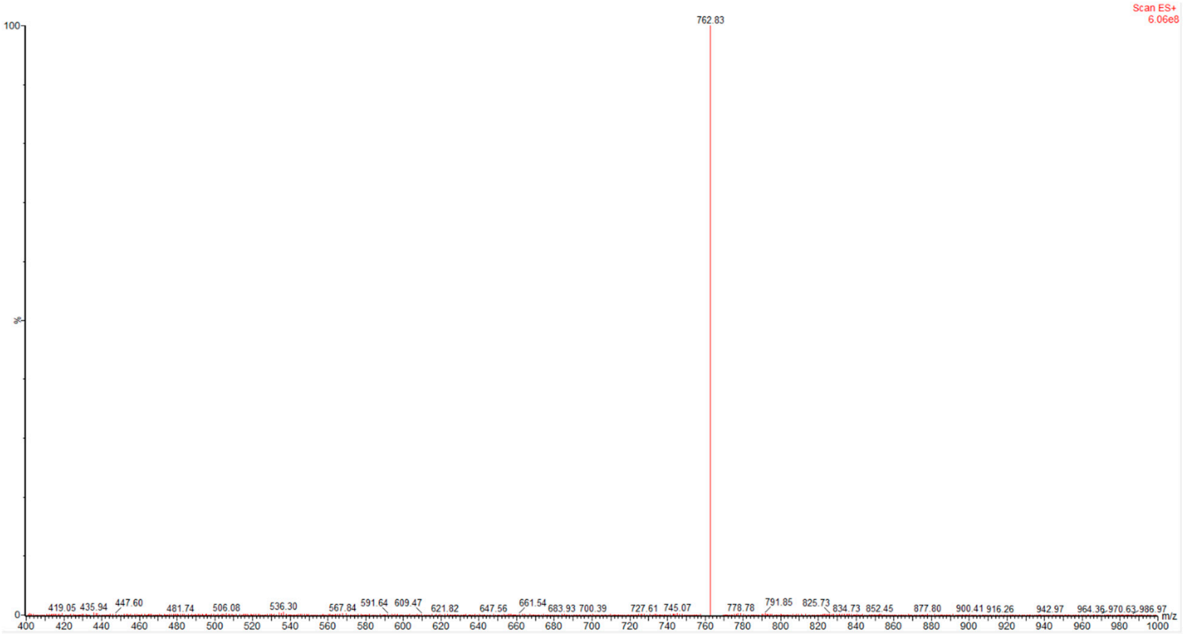

Figure S9. Mass spectrum of PP2.

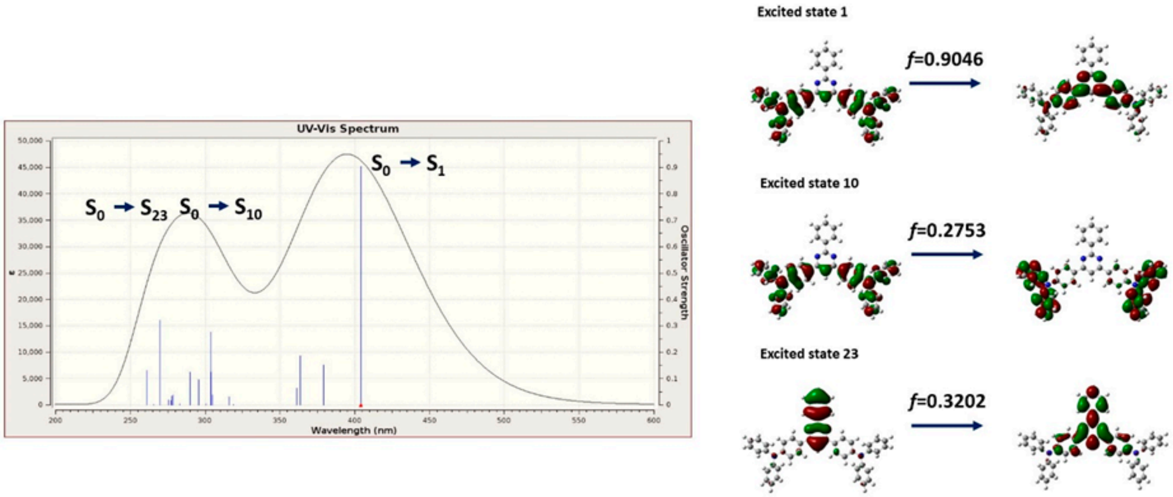

(**a**)

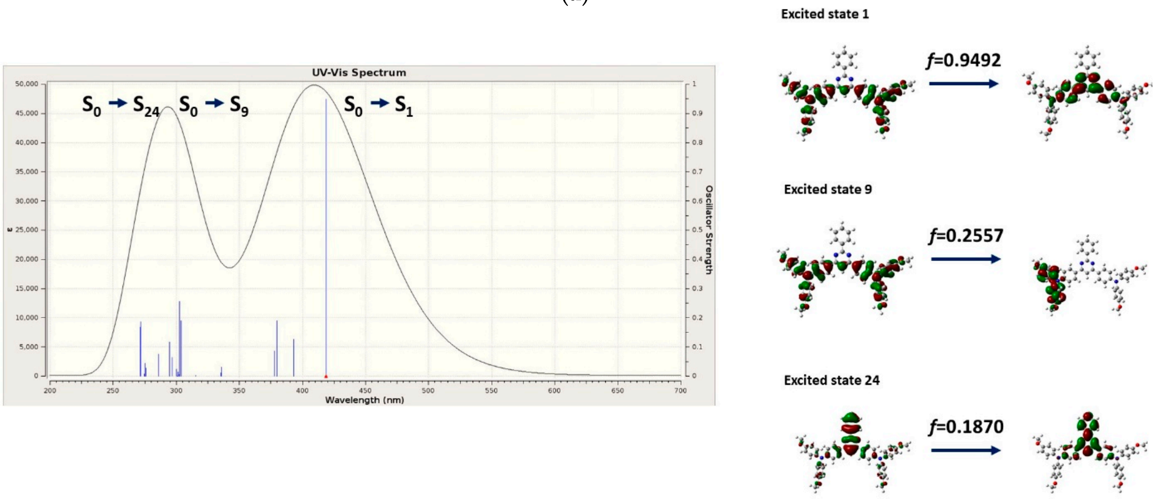

(**b**)

Figure S10. Theoretical UV spectra (dielectric constant evaluated ε = 6), and excited state geometries of compounds PP1 (a) and PP2 (b). (Grey colour: carbon; blue: nitrogen; red: oxygen; white: hydrogen).

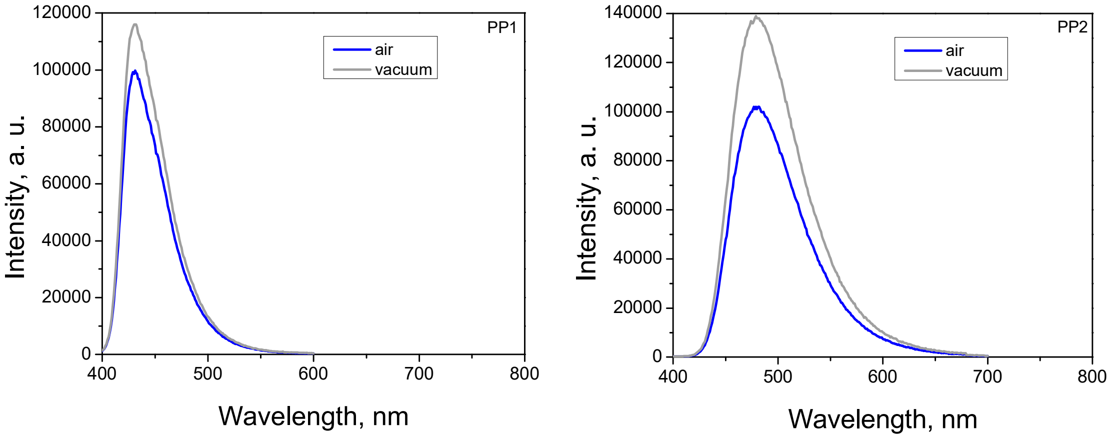

Figure S11. PL spectra of dilute toluene solutions of PP1 and PP2 after deoxygenation.

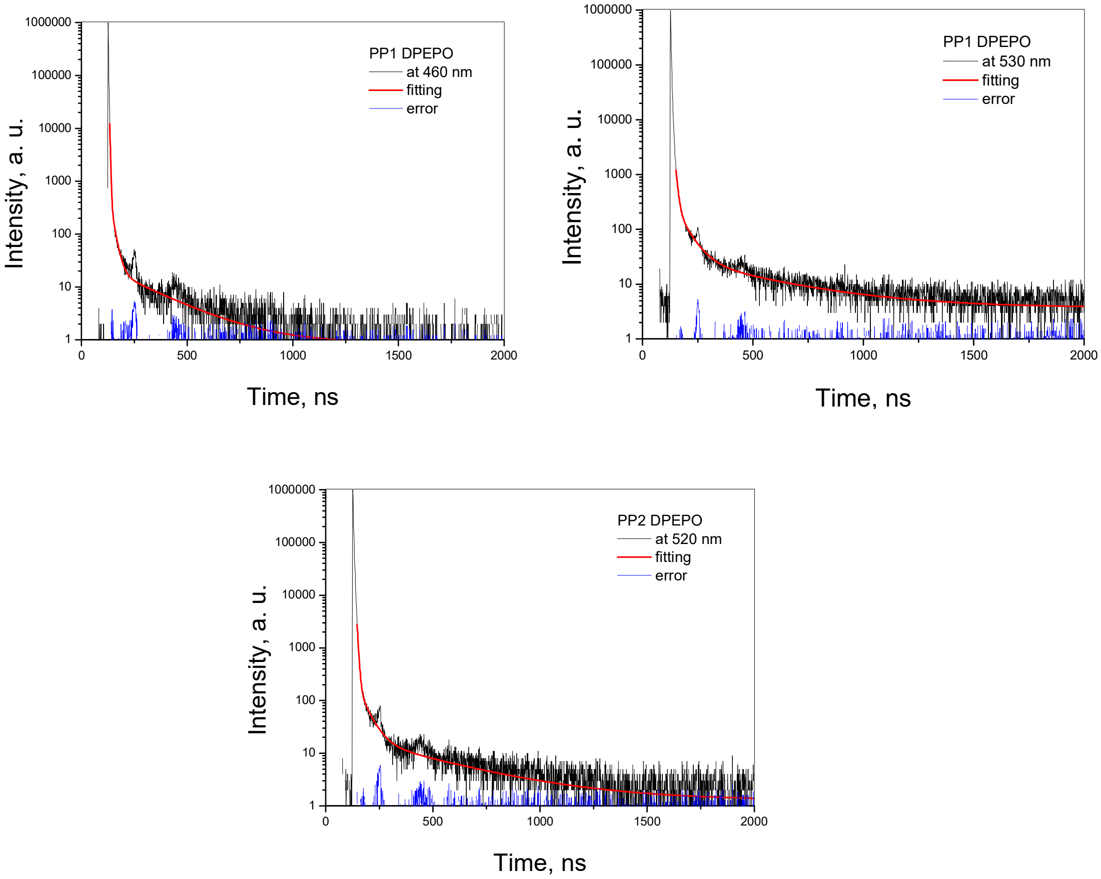

Figure S12. PL spectra of the films of PP1 and PP2 doped in DPEPO (20 wt.%) in vacuum at 300 K.

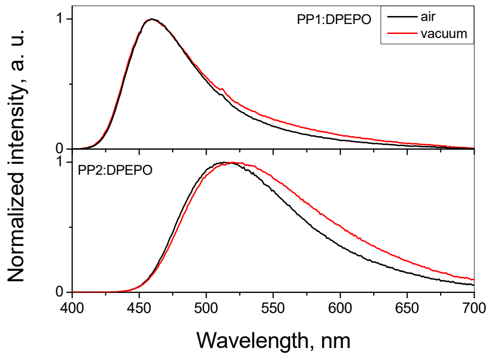

Figure S13. PL spectra of the films of PP1 and PP2 doped in DPEPO (20 wt.%) before and after removal of air.

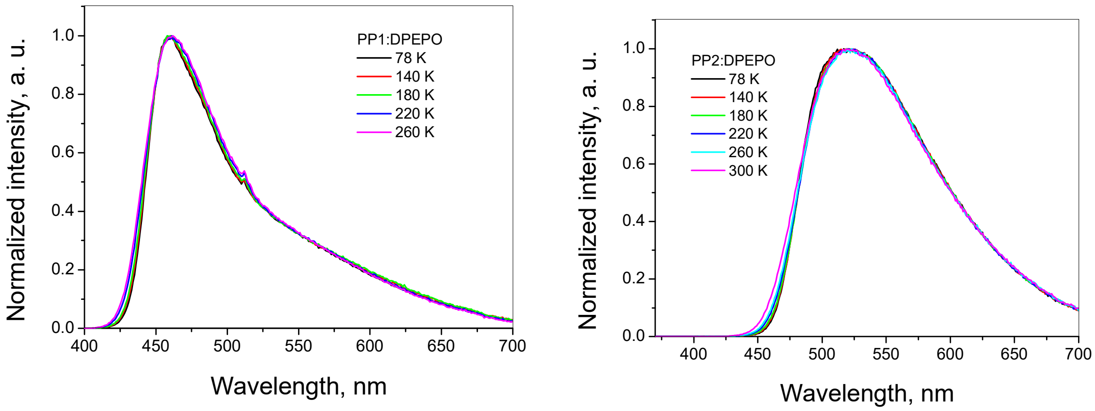

Figure S14. PL spectra of the films of PP1 and PP2 doped in DPEPO (20 wt.%) in inert atmosphere at different temperatures.

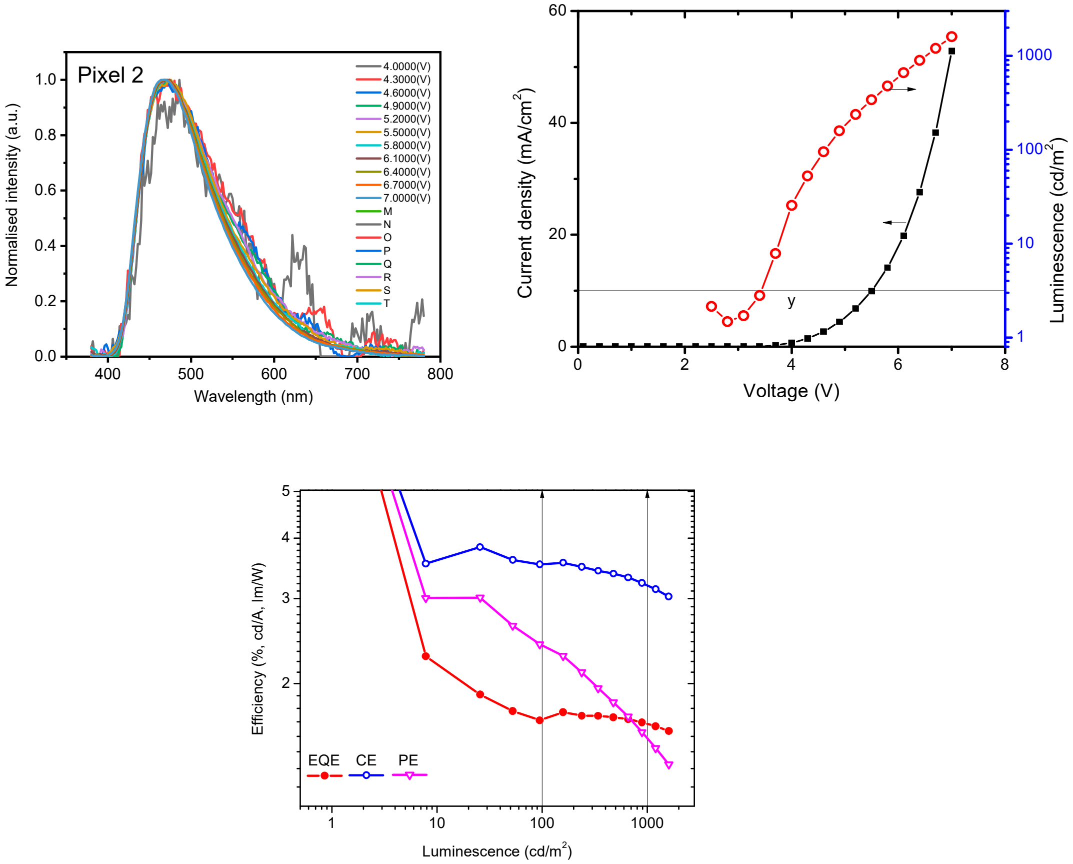

Figure S15. OLED A: EL spectra; luminescence and current density *vs* voltage correlations; current and power efficiency, EQE *vs* luminescence correlations.

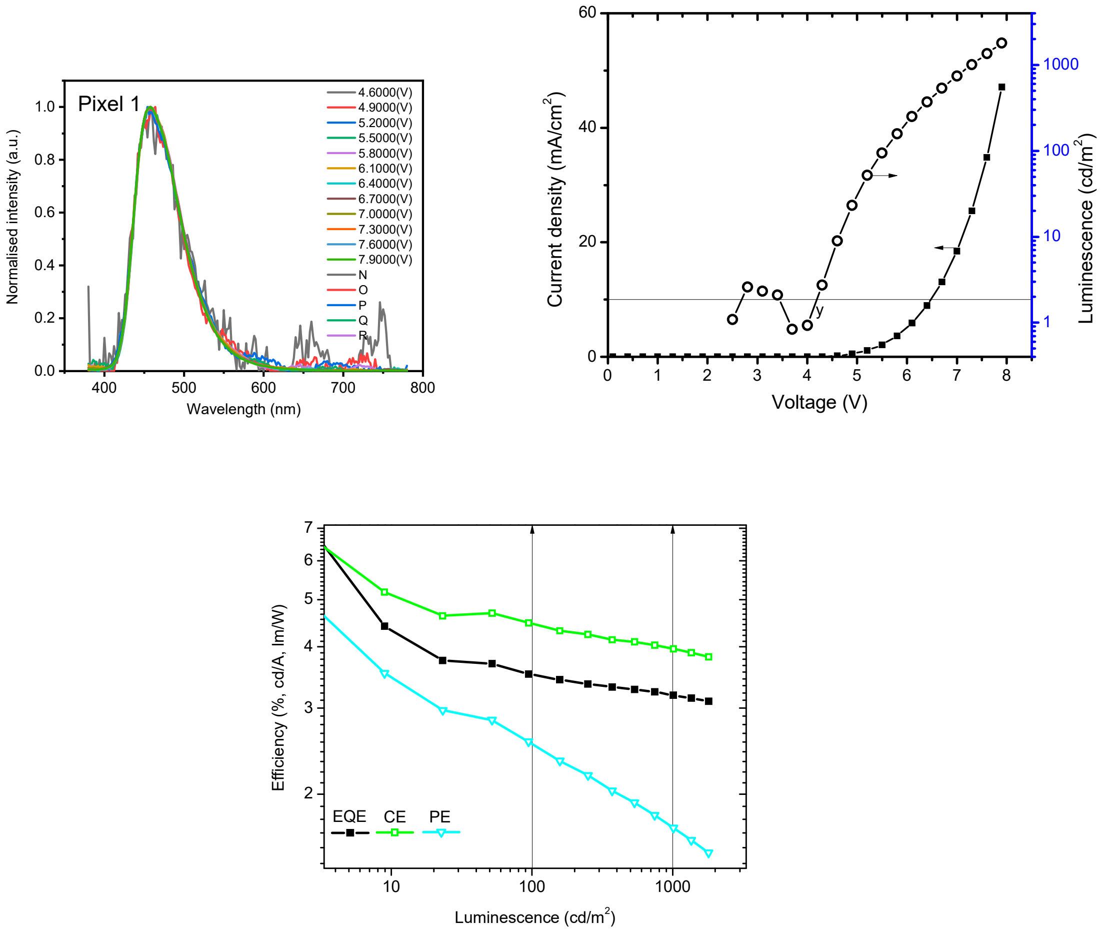

Figure S16. OLED B: EL spectra; luminescence and current density *vs* voltage correlations; current and power efficiency, EQE *vs* luminescence correlations.

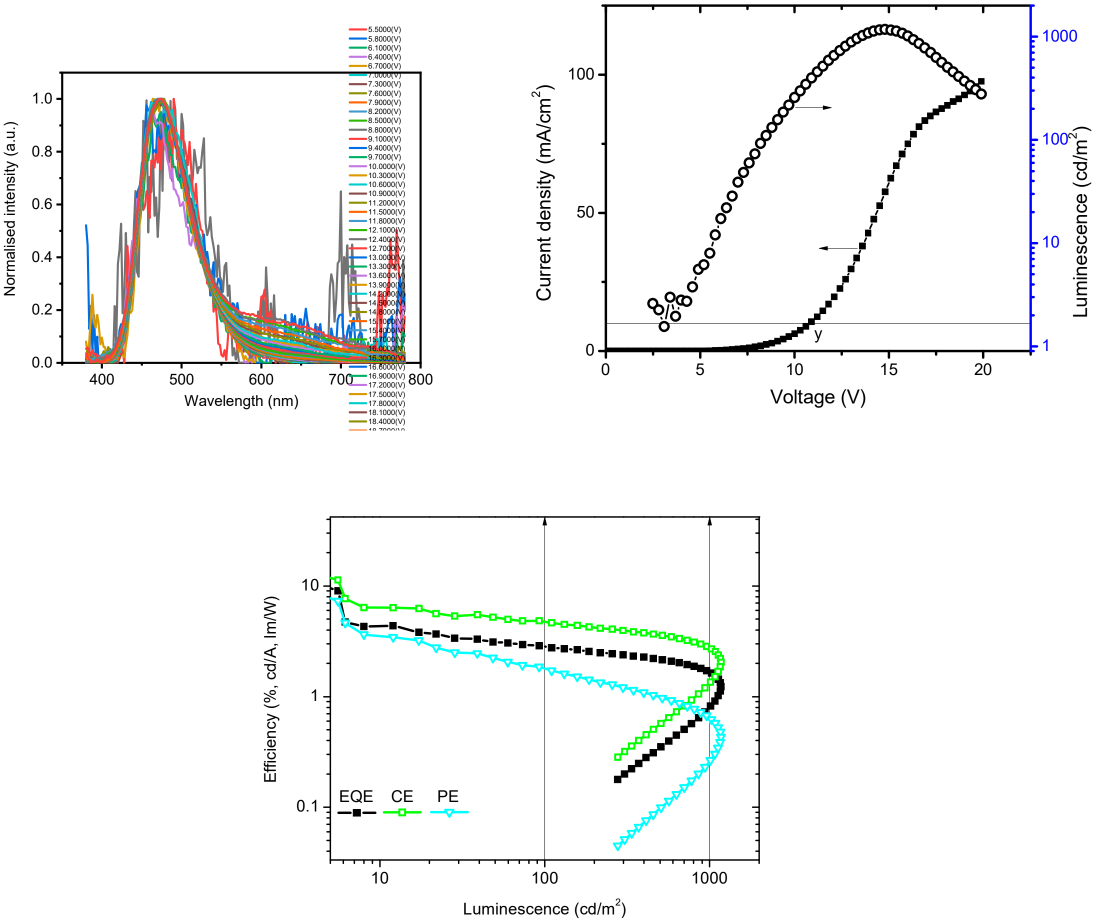

Figure S17. OLED C: EL spectra; luminescence and current density *vs* voltage correlations; current and power efficiency, EQE *vs* luminescence correlations.

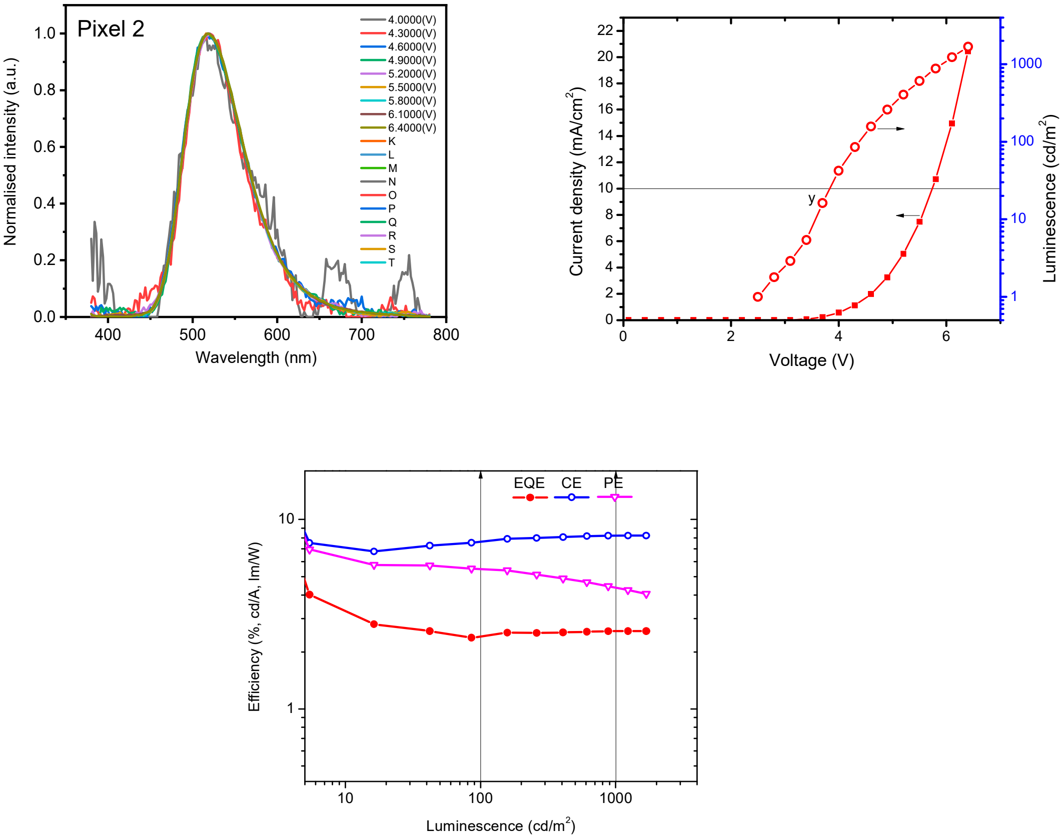

Figure S18. OLED D: EL spectra; luminescence and current density *vs* voltage correlations; current and power efficiency, EQE *vs* luminescence correlations.

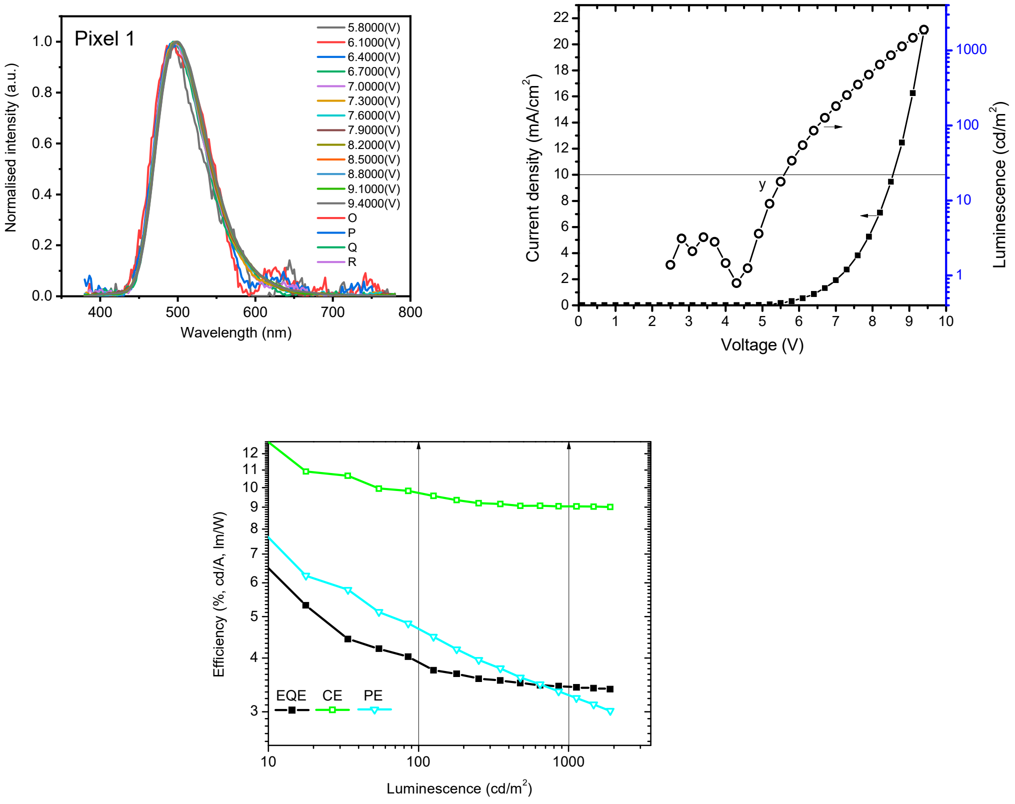

Figure S19. OLED E: EL spectra; luminescence and current density *vs* voltage correlations; current and power efficiency, EQE *vs* luminescence correlations.

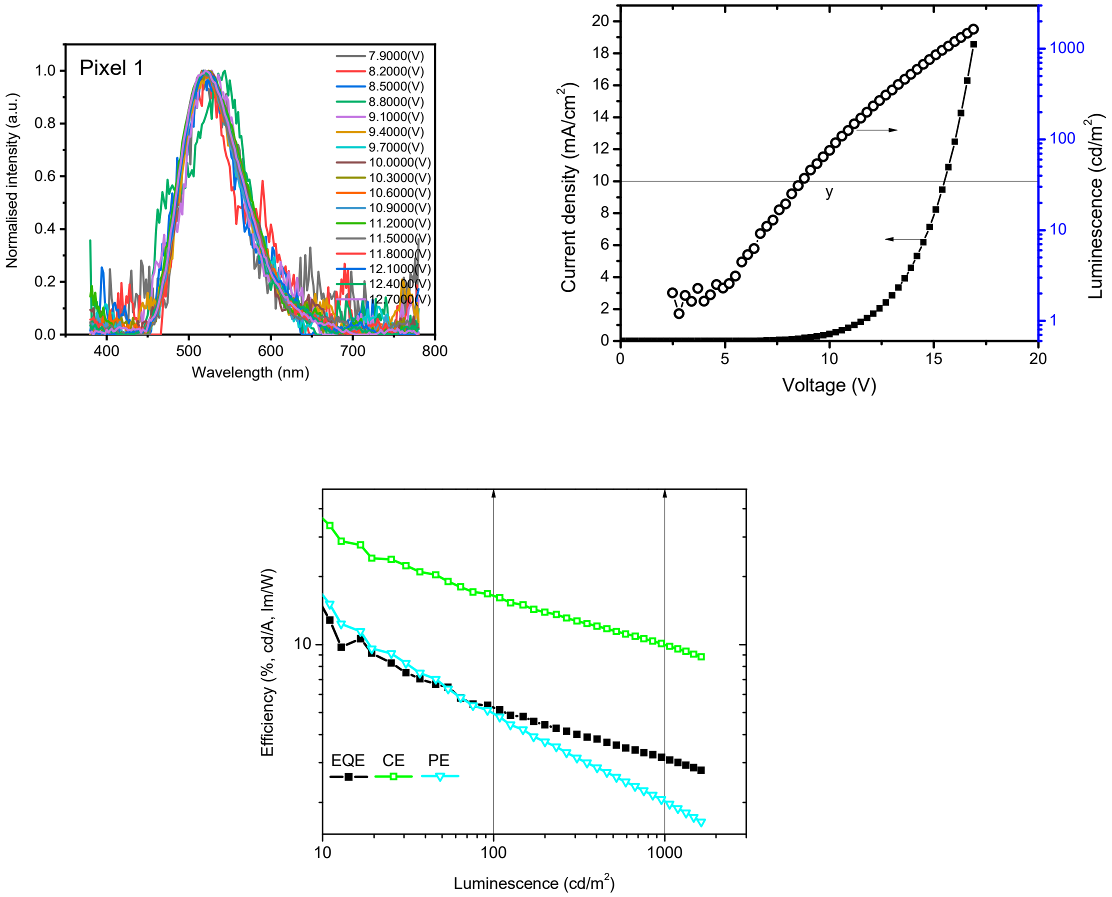

Figure S20. OLED F: EL spectra; luminescence and current density *vs* voltage correlations; current and power efficiency, EQE *vs* luminescence correlations.

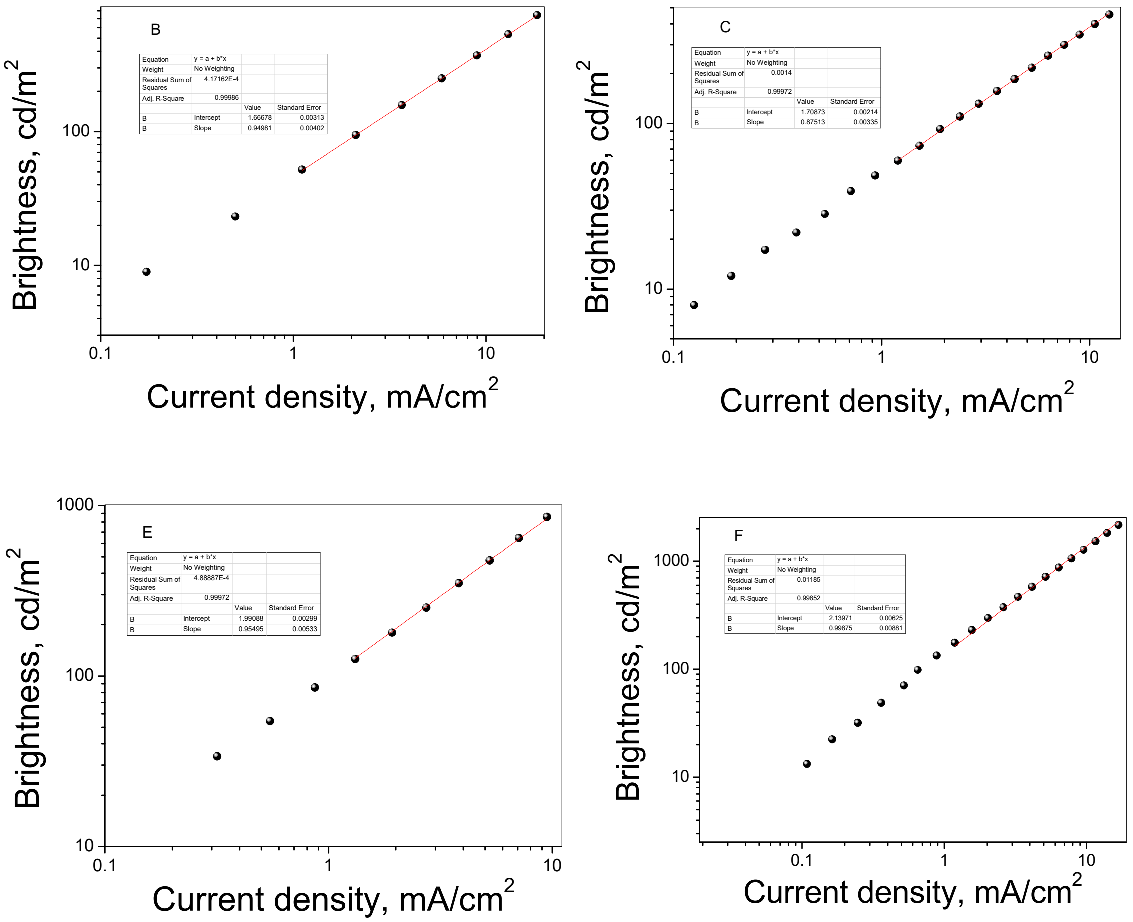

Figure S21. Correlation of brightness and current density for OLEDs B, C, E and F.

Table S1. Lifetime values of the exciton population derived from TREL decay curves of OLEDs related to the DF.

OLED	Lifetime, ms	χ^2
C	0.31 (11.13%), 2.08 (33.83%), 12.00 (55.04%)	1.177
E (5 wt.%)	5.78 (21.35%), 37.47 (78.65%)	1.013
F	5.47 (18.45%), 20.51 (52.76%), 75.73 (28.79%)	1.018

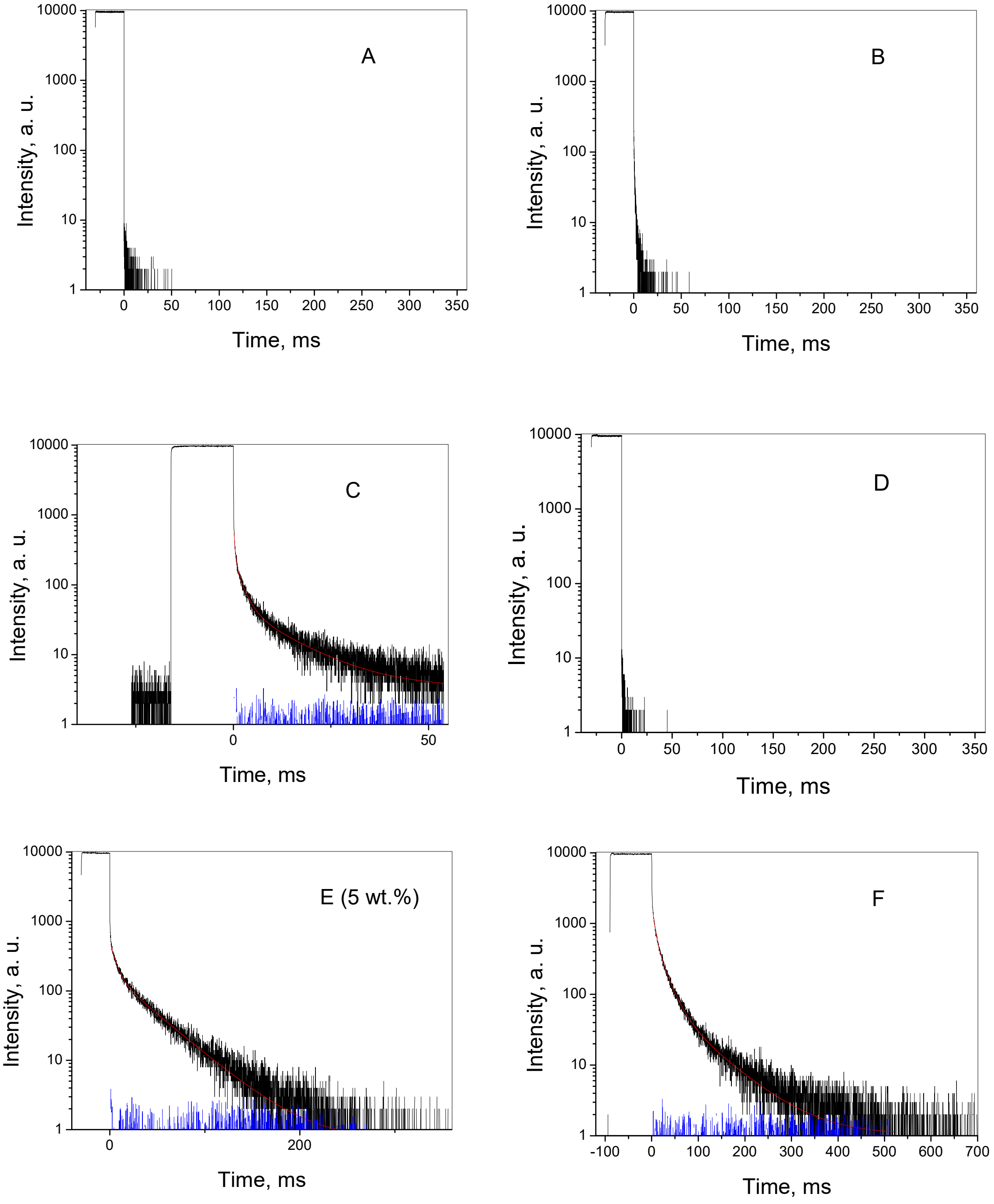

Figure S22. TREL decay curves of OLEDs.

References

51. Frisch, M.J.; Trucks, G.W.; Schlegel, H.B.; Scuseria, G.E.; Robb, M.A.; Cheeseman, J.R.; Scalmani, G.; Barone, V.; Petersson, G.A.; Nakatsuji, H.; et al. Gaussian 09, Revision A.02. *Gaussian, Inc. Wallingford CT* **2016**.

52. Hočevar, T.; Demčar, J. Computation of Graphlet Orbits for Nodes and Edges in Sparse Graphs. *J. Stat. Softw.* **2016**, *71*, 1-24 doi:10.18637/jss.v071.i10.
